# Development and validation of a multiplex UHPLC-MS/MS method for the determination of the investigational antibiotic against multi-resistant tuberculosis macozinone (PBTZ169) and five active metabolites in human plasma

**DOI:** 10.1371/journal.pone.0217139

**Published:** 2019-05-31

**Authors:** Dany Spaggiari, Vincent Desfontaine, Sandra Cruchon, Sylvie Guinchard, Anthony Vocat, Emilyne Blattes, Jeff Pitteloud, Lorenzo Ciullini, Carine Bardinet, Anton Ivanyuk, Vadim Makarov, Olga Ryabova, Thierry Buclin, Stewart T. Cole, Laurent A. Decosterd

**Affiliations:** 1 Laboratory & Service of Clinical Pharmacology, Department of Laboratories, University Hospital of Lausanne and University of Lausanne, Lausanne, Switzerland; 2 Global Health Institute, School of Life Sciences, EPFL, Lausanne, Switzerland; 3 Innovative Medicines for Tuberculosis (IM4TB), Lausanne, Switzerland; 4 Federal Research Center “Fundamentals of Biotechnology RAS”, Moscow, Russia; Medimmune, UNITED STATES

## Abstract

The emergence of *Mycobacterium tuberculosis* strains resistant to current first-line antibiotic regimens constitutes a major global health threat. New treatments against multidrug-resistant tuberculosis (MDR-TB) are thus eagerly needed in particular in countries with a high MDR-TB prevalence. In this context, macozinone (PBTZ169), a promising drug candidate with an unique mode of action and highly potent *in vitro* tuberculocidal properties against MDR *Mycobacterium* strains, has now reached the clinical phase and has been notably tested in healthy male volunteers in Switzerland. To that endeavor, a multiplex UHPLC-MS/MS method has been developed for the sensitive and accurate human plasma levels determination of PBTZ169 along with five metabolites retaining *in vitro* anti-TB activity. Plasma protein precipitation with methanol was carried out as a simplified sample clean-up procedure followed by direct injection of the undiluted supernatant for the bioanalysis of the six analytes within 5 min, using 1.8 μm reversed-phase chromatography coupled to triple quadrupole mass spectrometry employing electrospray ionization in the positive mode. Stable isotopically-labelled PBTZ169 was used as internal standard (ISTD), while metabolites could be reliably quantified using two unlabeled chemical analogues selected as ISTD from a large in-house analogous compounds library. The overall methodology was fully validated according to current recommendations (FDA, EMEA) for bioanalytical methods, which include selectivity, carryover, qualitative and quantitative matrix effect, extraction recovery, process efficiency, trueness, precision, accuracy profiles, method and instrument detection limits, integrity to dilution, anticoagulant comparison and short- and long-term stabilities. Stability studies on the reduced metabolite H_2_-PBTZ169 have shown no significant impact on the actual PBTZ169 concentrations determined with the proposed assay. This simplified, rapid, sensitive and robust methodology has been applied to the bioanalysis of human plasma samples collected within the frame of a phase I clinical study in healthy volunteers receiving PBTZ169.

## Introduction

Since *Mycobacterium tuberculosis* emerged some 70’000 years in Africa, it has never stopped to infect humans worldwide, making tuberculosis (TB) at present the deadliest infectious disease [1, 2]. According to recent WHO estimations, in 2016, 10.4 million people fell ill with TB, of whom over 4’500 people die every day. Although the TB mortality rate is globally falling at about 3% per year, the major concern is now the incidence of multidrug-resistant TB (MDR-TB) that is increasing, particularly in high TB burden countries (*e*.*g*., Russian Federation, China and India) [3]. As many as 490’000 patients have been diagnosed as being affected by MDR-TB in 2016 and an additional 110’000 TB people were identified as being resistant to rifampicin, the emblematic most effective first-line antitubercular drug so far. Globally, more than half million people could therefore be eligible to benefit from a new MDR-TB treatment, yet either not available or inaccessible to most of them. As recommended by WHO, a “short-course” TB combination therapy has to be the rule for treating the infection, and future drug candidates should be able to shorten treatment duration noticeably reducing the risks of treatment failure and disease relapse that arose after lack of compliance to prolonged treatment regimens, and *via* drug pressure, spread and increased prevalence of MDR-TB bacteria strains. Currently, several new or repurposed (or approved) anti-TB drugs are undergoing advanced phases of clinical development for *inter alia* MDR-TB treatment [4–8].

PBTZ169, also named macozinone, is a drug candidate with a new and unique mode of action, *i*.*e*., it covalently inhibits DprE1, a flavo-enzyme essential for the biosynthesis of key cell wall components of the pathogen [9–13]. This piperazinobenzothiazinone derivative was discovered through the FP6 “NM4TB” and FP7 “MM4TB” programs of the European Commission and optimized by medicinal chemistry from the lead BTZ043. Compared to BTZ043, PBTZ169 possess the following advantages: (i) easier chemical synthesis (no chiral center), (ii) low costs of goods and (iii) better pharmacodynamics properties [14, 15]. A remarkable *in vitro* effectiveness against *Mycobacterium tuberculosis* has been demonstrated for PBTZ169 with minimum inhibitory concentration (MIC) values below 0.0005 μg/mL, together with no general anti-bacterial activity [14]. In addition, PBTZ169 did not show antagonism with many TB therapeutic agents, both marketed or in development. Its additive effect with isoniazide, moxifloxacin, rifampicin, and synergistic effect with clofazimine or bedaquiline in preclinical models have opened new possibilities to develop a new regimen to treat MDR-TB [16–19].

*In vitro* metabolism studies using hepatocytes have demonstrated that PBTZ169 undergoes primarily hepatic phase I biotransformation into several metabolites. Interestingly, some metabolites retain the nitro group *(i*.*e*. pharmacophore) [20], and the hydroxyl- and oxo-metabolites of PBTZ169 have been found to still display anti-TB activities *in vitro* with appreciable MIC values. Recently, Kloss *et al*. [21] have reported the occurrence of a novel Macozinone metabolite H_2_-PBTZ169 that occurs *via* an unprecedented reduction process affording a Meisenheimer complex. As H_2_-PBTZ169 was reportedly highly unstable, easily reverting back to PBTZ169 upon air oxidation, which would spuriously affect the actual concentration of the parent drug PBTZ169, and Kloss *et al*. have therefore raised concerns about the ability to adequately quantify PBTZ169 in biological fluids.

An assay for PBTZ169 has been recently proposed [22], but allows the measurement of the parent drug only while active metabolites are likely to participate to the anti-TB activity and need therefore to be also quantified for comprehensive pharmacokinetics /pharmacodynamics analyses.

In this article, we aimed at developing and performing an extensive validation of a simplified, fast, robust and sensitive multiplex UHPLC-MS/MS method enabling the simultaneous quantification of PBTZ169 and five active metabolites in human plasma for its application in clinical studies.

## Material and methods

### Chemicals, reagents, compounds and other materials

**Fig 1:**
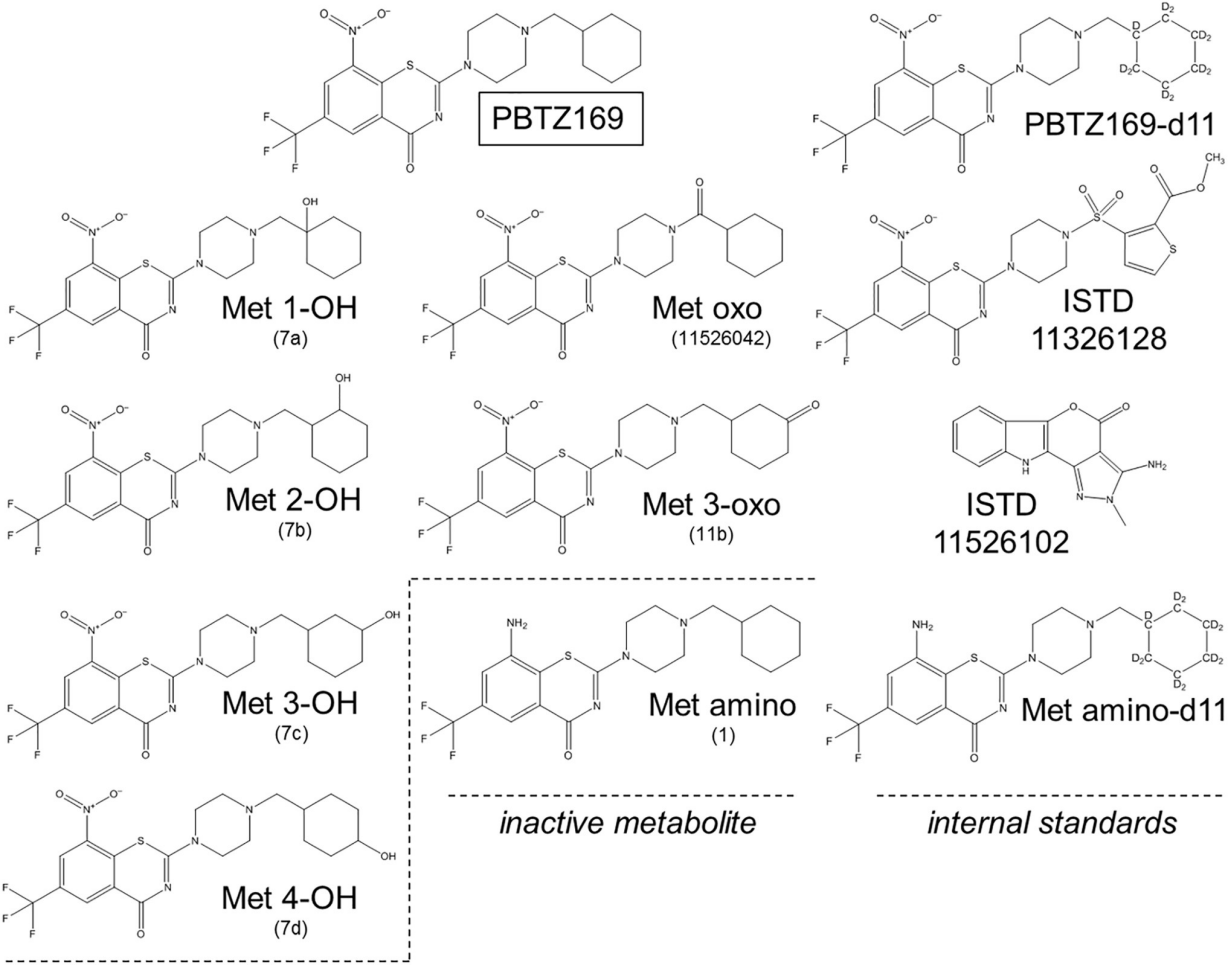
Chemical structures of PBTZ169, in vitro known metabolites and internal standards. In brackets, the names reported in the Investigator’s Brochure.

Acetonitrile (ACN), and methanol (MeOH) (Lichrosolv Reag. Ph. Eur. grade) and formic acid (FA, 98–100%) were of analytical grade and obtained from Merck (Darmstadt, Germany). Dimethyl sulfoxide (DMSO) was purchased at Panreac Applichem (Darmstadt, Germany). Ultrapure water was supplied by a Milli-Q Advantage A10 purification unit from Millipore (Bedford, MA, USA). PBTZ169 HCl (99.9%) was obtained from Aptuit (Oxford, United Kingdom), whereas active metabolites oxo (11526042, >99.8%), 1-OH (7a, >99.8%), 2-OH (7b, >99.8%), 3-OH (7c, >99.8%), 3-oxo (11b, >99.1%), inactive metabolite amino (1, >99.5%), internal standards PBTZ169-d11 (99.8%, isotopical purity 98.3%), Met amino-d11 (96.7%, isotopical purity 98.3%), dehydrogenated metabolite (H_2_-PBTZ169), compounds 11526102 and 11326128 were synthesized by the Federal Research Center “Fundamentals of Biotechnology RAS” (Moscow, Russia) (chemical structures are depicted in Fig 1). The 4-OH metabolite of PBTZ169 (7d, 99.6%) has been synthesized by the same manufacturer at a later stage of the present analytical development, and was used for metabolite peak identification only.

Blank human plasma from healthy donors (consenting collaborators of the laboratory) was used for *in vitro* drug stability studies and evaluation of anticoagulants matrix effect. According to Swiss regulation, the gift of a small volume (< 30 mL) of blood for laboratory purpose does not necessitate ethics committee approval. This has been confirmed by mails correspondence with our Institutional Ethics Committee. Otherwise, citrated plasma obtained from whole blood withdrawn from Vaquez patients at the occasion of their regular phlebotomy were used for the preparation of matrix-matched calibration samples. The use of otherwise discarded blood for the purpose of calibration samples preparation in the laboratory is in accordance with institutional regulations of our Hospital (Legal Department, April 4, 2017, Internal Note). Besides, two separate pooled lipemic plasma samples with triglycerides levels at 4.13 mmol/L and at 14.92 mmol/L (Cobas 8000 Hitachi, Roche Diagnostic, USA) were also used for method validation. Plasma was obtained by centrifugation (1970*g* (3000 rpm) for 10 min, +4°C, Hettich model Rotanta 460RF centrifuge; Tuttligen, Germany) after collection in trisodium citrate (3.2%) S-Monovette (Sarstedt, Nümbrecht, Germany) or in citrate phosphate dextrose adenine (CPDA-1) blood bag (CompoFlex, Fresenius Kabi, Oberdorf, Switzerland). For whole blood stability study, plasma was obtained by centrifugation (1970*g* (4550 rpm), 10 min, +4°C, Hettich model Mikro200R centrifuge, Tuttligen, Germany) of citrated whole blood in safe-lock polypropylene (PP) eppendorf tubes. For anticoagulants and serum comparison, whole blood was collected in Lithium-Heparin (16 I.U./mL blood), K_3_EDTA (1.6 mg/mL blood) and serum S-Monovette (Sarstedt, Nümbrecht, Germany), respectively. Serum was centrifuged after a 30-min whole blood clotting. All plasma and serum were stored in PP Falcon tubes (Corning, Mexico) at -20°C. For long-term stability study, spiked plasma was stored frozen (-20°and -80°C) in 1.8 mL CryoPure tubes (Sarstedt, Nümbrecht, Germany).

The phase 1a clinical study assessing the safety and pharmacokinetics of PBTZ169 in healthy volunteers (NCT03423030), was performed at the Service of Clinical Pharmacology of the Lausanne University Hospital and was approved on 06.09.2017 (2017–01241) by the Ethics Committee of the Canton of Vaud (CER-VD) and on 17.11.2017 (2017DR1171) by Swissmedic. All volunteers gave their written informed consent.

### Standard solutions preparation

#### PBTZ169, metabolites and internal standards stock solutions

Each compound was independently weighed and the corresponding powder dissolved in the required volume of DMSO to give a 1 mg/mL stock solution of the compound that was sonicated for 5 minutes. The stock solution used for PBTZ169 was corrected for salt form of the analyte by applying the weighing factor (0.926). Due to the high purity of the PBTZ169 and the five metabolites, no additional correcting factors were applied for preparing compounds stock solutions. Stock solutions were prepared in 5 mL PP tubes and stored at -20°C. The stability of these solutions was verified over a period of 6 months (mean bias < 8%) and of 9 months (mean bias < 9%)

#### Working solutions

PBTZ169 and metabolites mixture solutions. Two distinct working solutions (WS) including PBTZ169 together with its five active metabolites were prepared: one for calibration standards and one for validation standards/quality control (QC) samples. Working solutions including all the analytes at 100 μg/mL were obtained by adding the required volumes of compounds stock solutions (50 μL) to 200 μL of a DMSO/MeOH (1:1 *v/v*) mixture in safe-lock 1.5 mL PP tubes. Spiking solutions for calibration, validation standards and QCs at the appropriate concentrations were prepared by applying sequential dilutions in DMSO/MeOH (1:1 *v/v*) mixture starting from the dedicated WS in safe-lock 1.5 mL PP tubes and thoroughly mixed. Both working and spiking solutions were stored at -20°C or -80°C and their stabilities were verified over a period of 4 months at -20°C (mean bias < 6%) and 3 months at -80°C (mean bias < 7%).

Internal standards mixture solutions. Internal standards (ISTD) WS was prepared at 2000 ng/mL by adding the required volumes of three ISTD stock solutions, i.e. PBTZ-d11, compounds 11526102 and 11326128 (10 μL, each) to 4970 μL MeOH in a PP tube and stored at -20°C up to 3 months. Internal standards solution for plasma processing (plasma protein precipitation) was prepared in PP tube by diluting 50-fold in MeOH the internal standards WS to obtain a final concentration for all internal standards of 40 ng/mL. This solution was freshly prepared or stored at -20°C for 1 week and total volume prepared was adjusted as required.

#### Spiked plasma, serum or whole blood samples

All the spiked plasma, serum or whole blood used for method validation were obtained by diluting 20-fold the spiking solutions (50 μL) with blank biological matrices (950 μL). The total added volume was ≤10% of the biological sample volume respecting recommendations for bioanalytical method validation [23, 24].

### Human plasma processing procedure

Briefly, a 300 μL-volume of methanol containing internal standards at 40 ng/mL was added to 100-μL aliquot of human plasma (or serum) for plasma protein precipitation in 1.5 mL safe-lock PP tubes. The mixture was carefully vortexed and centrifuged at +4°C for 10 min at 18626*g* (14000 rpm, Hettich model Mikro200R centrifuge, Tuttligen, Germany). A 200μL-aliquot of limpid supernatant was directly transferred into a 2-mL glass vial with insert. Whole blood was processed as plasma after the centrifugation step described in Chemicals, reagents, compounds and other materials section.

### Liquid chromatography- tandem mass spectrometry

#### UHPLC-QqQ/MS instrumentation

The LC-MS/MS analyses were carried out using a Dionex Ultimate RSLC ultra-high-pressure liquid chromatography (UHPLC) system (ThermoFisher Scientific, San Jose, CA, USA). The instrument was equipped with a binary pump with a maximum delivery flow rate of 8 mL/min (up to 1034 bar), an autosampler including a flow-through needle and a column compartment thermostated at +40°C, the later being required for efficient separation of some closely related metabolites (see below). The UHPLC system was coupled through a diverter valve with a TSQ Quantiva triple quadrupole mass spectrometer (QqQ/MS) equipped with an Ion Max NG electrospray ionization source. The samples were stored at +5°C in the autosampler prior to and during the analyses. Data acquisition, treatment and instrument control were performed using XCalibur version 1.1 and Chromeleon version DCMS link (ThermoFisher Scientific, San Jose, CA, USA).

#### RPLC conditions

Optimal separation was performed with a Waters (Milford, MA, USA) Acquity UPLC HSS T3 column (1.8 μm, 2.1 mm × 50 mm) maintained at +40°C, and a flow rate of 600 μL/min. Gradient elution (solvent A, 0.2% FA in water; solvent B, ACN) was used according to the following program: isocratic step that holds 25% solvent B from 0 to 2.2 min, up to 90% solvent B in 1.5 min, held 90% solvent B to 4 min and column reconditioning at 25% solvent B to 5 min (total analysis time). The injection volume was 5 μL.

#### ESI-QqQ conditions

ESI was operated in the positive ionization mode applying static spray voltage set to 3500 V. Other optimized ESI source parameters were set as follows: the ion transfer tube and vaporizer temperatures at 400 and 300°C, respectively; sheath and auxiliary gas (nitrogen) flow rates at 80 and 7 (arbitrary units), respectively. The ESI probe depth was adjusted to the low (L), front (N°1) and central rotational positions on the Ion Max NG ion source.

The first (Q1) and third (Q3) quadrupoles operated with a mass resolution of 1.2 Da (i.e. m/z 1.2 FWHM). The cycle time was 0.2 s and peaks width for isocratically eluted compounds averaged 0.145 min, whereas PBTZ169 and Met-oxo peaks measured 0.06 min. Consequently, the number of MS/MS acquisitions was comprised within 43 and 18, respectively, which was adapted to the quantitative analysis purposes of the developed method. The Q2 collision gas (argon) pressure was 2 mTorr.

### Bioanalytical method validation

#### Selectivity

Matrix and cross-talk interferences. The selectivity of the method was established by the analysis of blank human plasma from five different donors (including two lipemic plasma samples) and two pooled regular plasma samples (n = 7), all processed with pure MeOH. UHPLC-MS/MS chromatograms were visually examined and compared for chromatographic integrity and potential interferences from processed matrices.

Selectivity was additionally evaluated by analyzing the highest calibration standard sample (2000 ng/mL) processed with pure MeOH and a blank human plasma processed with only internal standards solution at 40 ng/mL, to identify potential (i) interferences affecting co-eluting analytes and internal standards due to the MS-based cross-talk phenomenon or (ii) presence of isobaric impurities.

#### Matrix effect, extraction recovery and process efficiency

Qualitative evaluation of matrix effect. The potential impact of endogenous components present in human plasma on ionization process of PBTZ169, metabolites and ISTDs was assessed qualitatively by individual post-column infusion (10 μL/min) of the analytes (at 500 ng/mL in MeOH) directly into MS spectrometer during the UHPLC-MS/MS analysis of blank human plasma from 7 different donors (including two lipemic plasma) and three pooled regular plasma (n = 10), all processed with pure MeOH (*i*.*e*. not containing ISTDs) [25]. The total ion current LC-MS/MS signals for each analyte (*i*.*e*. sum of all MS/MS transitions) were visually examined to check for any signal perturbation (drift or shift) of the MS/MS signal at the analytes’ retention times [26].

Quantitative assessment of matrix effect, extraction recovery and process efficiency. Matrix effects (ME), extraction recoveries (ER) and process efficiencies (PE) for PBTZ169, five active metabolites and internal standards were quantitatively assessed according to Matuszeswski’s procedures as well as to the recommendations of the EMEA/FDA [27, 28]. Three sets of standards samples at three concentration levels (10, 100 and 1000 ng/mL) were prepared: set A included three matrix free samples (neat standard in water/MeOH 1:3), set B included seven post-extraction spiked human plasma, and set C had seven pre-extraction spiked plasma. For set B and C, five individual regular plasma from healthy donors and two pooled lipemic plasma were used and extractions were done in duplicates. For calculations, the mean peak area of the three determinations obtained for set A was used as reference, whereas for set B and C, each replicate was taken into account as an independent measure (*i*.*e*. n = 10 for regular plasma and n = 4 for lipemic plasma). The variability of the parameters between plasma was evaluated for regular and lipemic plasma separately and was expressed as relative standard deviation (RSD). ME was calculated by expressing the analyte peak area obtained in post-extraction spiked human plasma as percentage of the analyte peak area obtained in matrix free samples (water/MeOH 1:3) (B/A in %) and results were reported as deviations (%) [29]. ER was estimated by expressing analyte peak area measured in pre-extraction spiked plasma as percentage of that obtained in post-extraction spiked human plasma (C/B in %). PE, which takes into account matrix effect and extraction recovery, was assessed by expressing the analyte peak area in pre-extraction spiked plasma as percentage of that measured in matrix free samples (C/A in %). The internal standards-normalized ME, ER and PE (i.e. IS-nME, IS-nER, IS-nPE) were calculated using the analyte/ISTD peak area ratios.

#### Trueness, precision and accuracy profiles

Trueness and precision of the multiplex UHPLC-MS/MS method for quantitative determination of PBTZ169 and five active metabolites were assessed over three separate days (j = 3). Pooled blank regular matrix from two different donors was used for preparation of calibration and validation standards. For calibration standards in plasma, nine concentration levels (k) in triplicates (n = 3) were independently prepared each validation day at the following concentrations: 0.1, 0.2, 0.5, 1, 2, 50, 500, 1000, 2000 ng/mL. Calibration ranges retained for quantification were as follows: 0.1–2000 ng/mL (k = 9) for PBTZ169, 0.2–2000 ng/mL (k = 8) for Met 3-OH and Met 3-oxo, and 0.5–2000 ng/mL (k = 7) for Met oxo, Met 1-OH and Met 2-OH. For validation standards in plasma, eight concentration levels (k) in triplicates (n = 3) were independently prepared each validation day at the following concentrations: 0.1, 0.2, 0.5, 1, 2, 150, 750, 1500 ng/mL. Validation standards were quantified as follows: 0.1–1500 ng/mL (k = 8) for PBTZ169, 0.2–1500 ng/mL (k = 7) for Met 3-OH and Met 3-oxo and 0.5–1500 ng/mL (k = 6) for Met oxo, Met 1-OH and Met 2-OH. To confirm the LLOQ values, each analyte had at least two low validation sample concentrations (one at LLOQ and one at 2-3x LLOQ). The concentration ranges have been selected according to anticipated patients’ plasma values, based on pre-clinical data.

The trueness, for systematic errors estimation, was expressed as the ratio between the measured mean and reference nominal concentration, while precision, for random errors assessment, was estimated with variances of repeatability (intra-day variances) and intermediate precision (sum of intra-day and inter-day variances), both calculated as described in the SFSTP reports [30–34]. According to recommendations, precision parameters were finally reported as RSD based on the reference nominal value at each concentration level [35, 36].

Accuracy profiles were used to evaluate the total error of the developed bioanalytical method by integrating both systematic and random errors. This applied approach is based on β-expectation tolerance interval (ETI) and represents the concentrations range where β% of future results is expected to be found [37, 38]. For ease of interpretation, accuracy profiles were presented as total relative error (%) calculated from reference nominal concentration. For PBTZ169, accuracy profile in absolute concentrations was used to obtain a better estimation of the LLOQ.

#### Linearity of trueness

Linearity of the trueness is required and defines the ability of the method to give quantitative results directly proportional to known nominal analyte concentrations within the validated range. This parameter was determined by ordinary linear square regression using the data plots reporting experimental concentrations as function of nominal concentrations (for the three days of validation).

#### Integrity to dilution

The ability to dilute samples containing PBTZ169 and metabolites at concentrations above the ULOQ was demonstrated by performing six replicates 10-fold dilutions (with different human plasma donor) of human plasma samples spiked at 5000 ng/mL. Concentrations of analytes in these matrix dilution samples were determined with the validated regression model for quantification. Back-calculated concentrations of the non-diluted samples (n = 4) were also extrapolated.

#### Carryover

For PBTZ169 and metabolites, carryover was assessed by injection of one or more blank solvent (MeOH) after highest calibration standard sample (2000 ng/mL).

#### Method and instrument detection limits

The limit of detection (LOD) for PBTZ169 and its five active metabolites in human plasma was determined in three different human plasma as the lowest analyte concentration possessing a signal-to-noise (S/N) ratio equal and greater than 3. For peak and noise intensities measurements, Genesis algorithm (part of Xcalibur software) was applied for peak tracking and noise was processed as Peak-to-Peak within at least 30 seconds before or after the peak (the most intense was selected).

The instrument detection limit (IDL) is the minimum amount of analyte required producing a LC-MS signal (peak area) that is statistically distinguishable from the background noise level within a specified confidence level [39–41]. This approach is based on statistically valid measurement that eliminates the uncertainty and variability associated with S/N ratio calculations allowing reliable performances comparison between different LC-MS instruments [42–44]. The IDL measurement for the TSQ Quantiva in the optimized LC-MS/MS conditions was assessed in three different post-extraction spiked (2.5% of matrix volume) human plasma using ten replicate injections of 2–5 folds the expected detection limits (0.05 ng/mL for all analytes). The IDL values for PBTZ169 and its active metabolites were calculated by applying the following formula: IDL = 2.821 x (RSD/100) x injected amount, where 2.821 is the confidence factor t_α_ coming from the one-side t-Student distribution table for n-1 degree of freedom (n is the number of replicates) and 99% confidence level, whereas RSD is the peak area precision obtained for ten replicate injections.

#### Stabilities

Bench-top stability experiments. Stabilities of PBTZ169 and its active metabolites in plasma as well as in post-preparative samples stored at +4°C and at room temperature (RT) were assessed for QC at 2, 150 and 1500 ng/mL (n = 3) by comparing the mean concentration of samples stored for 24 hours against concentrations of samples prepared at t_0_ and stored for 24 hours at -20°C.

Stability in citrated whole blood was evaluated at RT, +4°C and in ice for 1, 2 and 4 hours at two different (low and high) QC concentrations (10, 100 ng/mL in ice; 20, 200 ng/mL at +4°C; and 50, 500 ng/mL at RT). Deviations of the mean concentrations (n = 3) of samples measured at each time points from the mean concentrations (n = 3) of samples prepared at t_0_ were calculated.

Stability in spiked human plasma after three freeze-thaw cycles from -20°C to RT was assessed using calibration standard samples in duplicate while stability after three freeze-thaw cycles from -80°C to RT was assessed with QC samples at 2, 150 and 1500 ng/mL in triplicate. In both cases, samples underwent 1h-freeze and 1h-thaw processes three times. The mean concentration of samples after three freeze-thaw cycles was compared to the corresponding concentrations of freshly prepared samples.

Long-term stability in plasma. Stability in citrated plasma stored at -20°C and -80°C in suitable tubes for long-term storage (1, 3, 6 and 12 months) was evaluated at 1, 10, 100 and 1000 ng/mL. Deviations of the mean concentrations (n = 3) of samples measured at each time points from the nominal concentrations were calculated.

#### Anticoagulants and serum comparison

A head-to-head comparison between different anticoagulants (*i*.*e*., citrate, EDTA and heparin) and serum was performed using whole blood from the same human donor. Bias (%) and precision (RSD) for quantification of PBTZ169 and active metabolites in plasma with different anticoagulants (EDTA and heparin) and in serum by using validated citrated plasma calibration were assessed with QC samples (n = 3) spiked at 10, 150 and 1500 ng/mL. Additionally, EDTA plasma spiked at calibrators levels were also quantified using reference calibration set prepared in citrated plasma.

## Results and discussion

### Analytical method development

**Fig 2:**
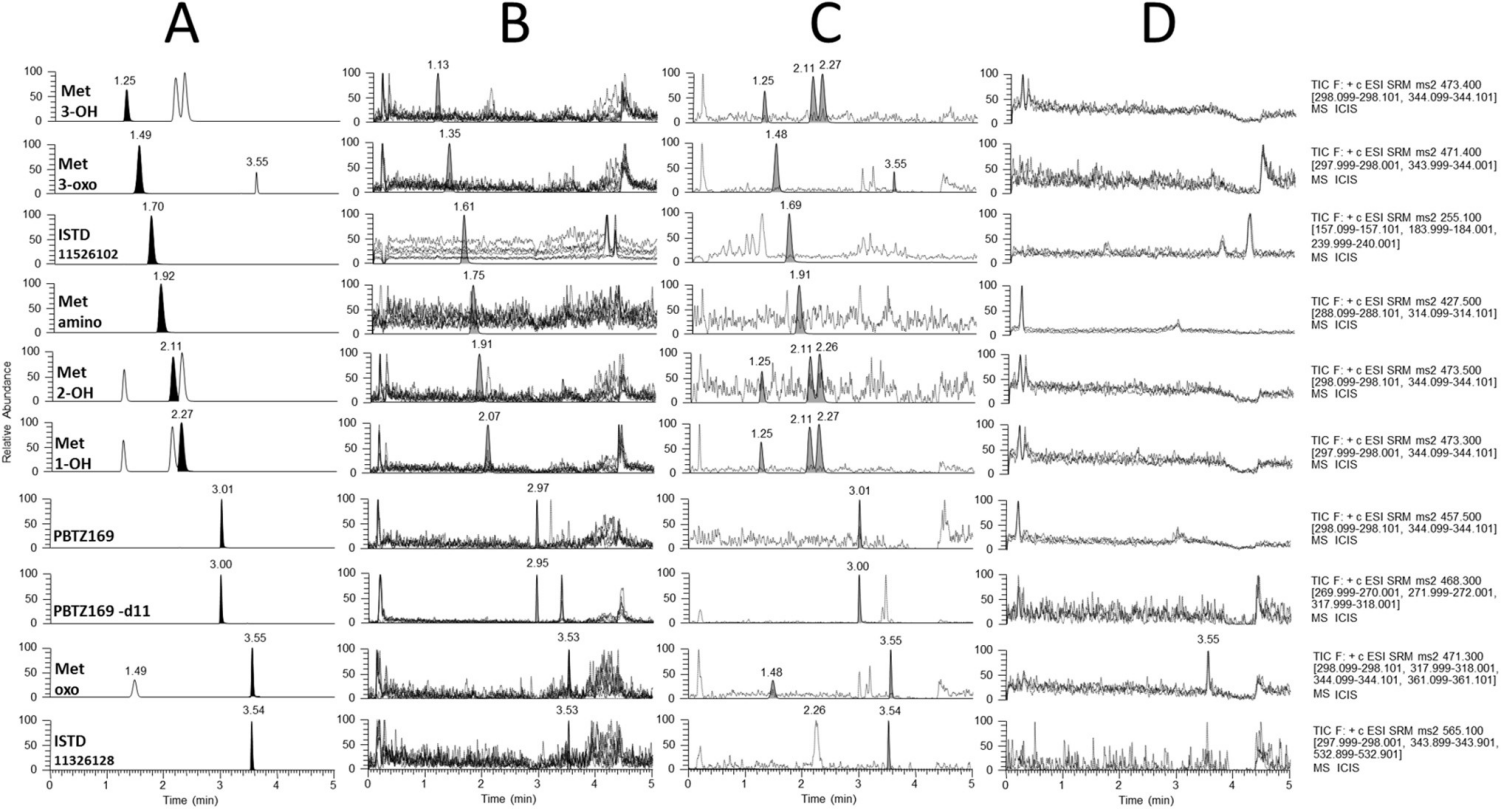
(A) UHPLC-MS/MS separation of PBTZ169, six in vitro known metabolites and internal standards in spiked human plasma (calibration sample at 500 ng/mL). Each targeted compound is reported as a black chromatographic peak at the selected m/z transition (i.e. to distinguish them from position isomers signals). (B) Selectivity for matrix interferences: overlaid UHPLC-MS/MS profiles (dotted lines) of methanolic extracts from ten blank human plasma. (C) Selectivity for analytical interferences: UHPLC-MS/MS profiles for checking mutual interferences between analytes and ISTD. The background profiles (dotted lines) reported in the analytes LC-MS/MS traces are obtained after precipitation of a human blank plasma with processed with only ISTD solution at 40 ng/mL, whereas background profiles depicted in the ISTD LC-MS/MS traces are obtained after precipitation of the highest calibrator level (2000 ng/mL) with pure MeOH. For (B) and (C), retention times and chromatographic LC-MS/MS profiles of PBTZ169, active metabolites, Met-amino and ISTD obtained during experiments were superimposed for interpretation. (D) Carryover: overlaid UHPLC-MS/MS profiles (n = 3) obtained for the first injection of blank solvent (MeOH) after the highest calibration sample (2000 ng/mL).

Overall analytical efforts aimed at allowing a comprehensive pharmacokinetic characterization of not only the parent drug, but also of its as yet identified *in vivo* pharmacologically active metabolites, as recommended by FDA guidelines on drug safety [45]. Several PBTZ169 metabolites observed *in vitro* during hepatocytes incubation experiments have been also identified in various animal species (mice, rat, dog) as well as in human [46]. These metabolites have been synthesized chemically and comprise five active metabolites, namely 1-OH, 2-OH, 3-OH, oxo, 3-oxo and one inactive metabolite amino (Fig 1). The synthesized metabolite 4-OH, available at a later stage of method validation, was only used for chromatographic peak identification. As shown in Fig 1, some metabolites are positional isomers (*e*.*g*., monohydroxylated metabolites, with the same molecular formula and exact mass) with closed physicochemical properties (S1 Table), precluding their unambiguous characterization using mass spectrometry only and making mandatory a chromatographic separation for accurate quantitation. The order of elution of analytes has been formally established by individual LC-MS/MS injection of each compound. The most critical pair to resolve was the isomeric Met 1-OH/Met 2-OH pair, and Met 3-OH/Met 4-OH pair, for which a satisfactory chromatographic separation (resolution >1.2) for reliable peaks integration was only obtained isocratically using conventional mobile and stationary phases. Of note, maintaining the UHPLC column in a temperature-controlled oven at +40°C was found to play a critical role to achieve the required selectivity for those isomeric hydroxylated pairs separation, besides being able to reduce the starting high column-backpressure to approximately 350 bars. Due to the multiple pro-chiral centers on the cyclohexyl moiety of PBTZ169, its hydroxylated metabolites 2-OH, 3-OH and 4-OH (the later synthesized only at a later stage of analytical development) may occur as diastereoisomers. The synthetic hydroxylated metabolite standards were available as diastereoisomers mixture that gave a single peak for Met 2-OH, Met 3-OH, while the two Met 4-OH diastereoisomers could be separated with the same applied chromatographic elution program (see Pharmacokinetics application section). As depicted in Fig 2A, an efficient separation of pure standard of PBTZ169 and the first six available metabolites was achieved in less than 4 min with good peak shape for all compounds. In addition, the base-line separation of the Met 3-OH and Met 4-OH is shown thereafter in the chromatographic profile of a volunteer plasma sample.

As depicted in the S1 Fig, monitored endogenous phospholipids were chromatographically eluted at retention times that do not interfere with the mass spectrometric detection of PBTZ169 and metabolites, reducing the risk of relevant matrix effect [47–49]. Stable isotope-labeled PBTZ169-d11 and Met-amino-d11 were synthetized as ISTD, while foridentifying suitable ISTDs for the pharmacology active metabolites oxidized onto the cyclohexyl site (difficulty amenable to similar labeling approaches), we have carried out an extensive screening of the new-chemical-entities’ library of the Global Health Institute (EPFL, Lausanne, Switzerland) [50]. Seventy-seven compounds were selected mainly according to their molecular structures similarities and physico-chemical properties, including PBTZ169- (35%), BTZ043- (15%) derivatives and chemical entities (50%).

By applying the optimized chromatographic separation program, seven compounds eluted within the isocratic step and retention times ranged from 1.0 to 2.5 min, *i*.*e*., a retention interval in which the four actives metabolites, namely 3-OH, 3-oxo, 2-OH and 1-OH, were included. The 60% of the screened compounds had retention times between 2.8 and 3.7 min, with five compounds co-eluting with Met oxo while the remaining 30% eluted during the column rinsing step or were not detected. The majority of the compounds showed sensitivity issues, or instability during prolonged analytical sequence, and/or co-elution with *in vivo* abundant unknown metabolites. Finally, only two compounds (chemical structure reported in Fig 1) were found suitable to be employed as ISTD for quantitative measurements of oxidized PBTZ169 metabolites in human plasma: compound 11526102 (t_R_ 1.7 min) was associated to metabolites 1-OH, 2-OH, 3-OH, 3-oxo, while compound 11326128 (PBTZ169 derivative eluting at 3.55 min) was associated to Met oxo.

For PBTZ169 and metabolites, product ions spectra were acquired in positive ESI mode and are reported in S2 Fig. At least two product ions were identified and selected for each compound to ensure maximal sensitivity and higher selectivity by applying MRM detection. Of note, for PBTZ169 as well as metabolites 1-OH, 2-OH, 3-OH, 3-oxo, the combination of SRM signals obtained for product ion m/z 298 and 344 was used for quantification due to the improvement of sensitivity (S/N approximately 20% higher) compared to the single SRM. The characteristic precursors [M+H]^+^ to product ion transitions as well as ion ratios are reported in Table 1.

**Table 1:** Molecular formula and mass, MS/MS parameters and typical retention times for the analysis of PBTZ169, *in vitro* known metabolites and internal standards.

*Compound*	Formula	Molar mass	Precursor ions [M+H]^+^	Product ions [M+H]^+^	Ion ratio^a^	CE	RF Lens	Typical t_R_^b^
		*g/mol*	*m/z*	*m/z*		*V*	*V*	*min*
PBTZ169	C_20_H_23_F_3_N_4_O_3_S	456.5	457.5	344.1	1	23	103	3.0
				298.1	0.68	36		
*Active metabolites*								
Met 1-OH	C_20_H_23_F_3_N_4_O_4_S	472.5	473.3	344.1	1	23	93	2.3
				298.0	0.82	42		
Met 2-OH	C_20_H_23_F_3_N_4_O_4_S	472.5	473.5	344.1	1	26	104	2.1
				298.1	0.74	40		
Met 3-OH	C_20_H_23_F_3_N_4_O_4_S	472.5	473.4	344.1	1	26	107	1.2
				298.1	0.67	39		
Met 3-oxo	C_20_H_21_F_3_N_4_O_4_S	470.5	471.4	344.0	1	26	107	1.5
				298.0	0.86	39		
Met oxo	C_20_H_21_F_3_N_4_O_4_S	470.5	471.3	361.1	1	19	94	3.5
				344.1	0.12	30		
				318.0	0.08	33		
				298.1	0.11	43		
*Inactive metabolite*								
Met amino	C_20_H_25_F_3_N_4_OS	426.5	427.5	314.1	1	26	160	1.9
				288.1	0.34	26		
*Internal standards*								
PBTZ169-d11	C_20_H_12_D_11_F_3_N_4_O_3_S	467.6	468.3	318.0		26	119	3.0
				272.0		39		
				270.0		50		
Met amino-d11	C_20_H_14_D_11_F_3_N_4_OS	436.7	438.3	288.0		30	100	1.8
				314.1		28		
11326128	C_19_H_15_F_3_N_4_O_7_S_3_	564.5	565.1	532.9		24	135	3.5
				343.9		31		
				298.0		24		
11526102	C_13_H_10_N_4_O_2_	254.2	255.1	240.0		23	101	1.7
				184.0		33		
				157.1		35		

^a^ Calculations are based on peak area and report the mean of ion ratios measured in calibration levels.

Peak identity is confirmed when ion ratio is within ±20% in real human sample.

^b^ Minor changes in retention times are possible, in particular for compounds eluted isocratically.

The ISTD concentrations were selected to have peak intensities relative to target analytes concentrations that would provide satisfactory ISTD-normalized response functions.

The ESI source parameters are reported in S3 Fig. They were optimized to maximize ionization efficiency at high mobile phase flow rate with the best compromise in terms of sensitivity for PBTZ169 and metabolites while minimization of background noise.

### Method validation

#### Selectivity and carryover

As shown in Fig 2B, no significant interferences from endogenous plasma components were observed at the retention time of the PBTZ169, metabolites and ISTD. For Met oxo, a single lipemic plasma was found to interfere with the transition used for quantification (*m/z* 471 to 361) albeit having less than 30% of the LLOQ signal. Yet, to minimize the risk of interferences that would impact the Met oxo metabolite detection in patient’s samples, three additional characteristic product ions were used in the MS/MS acquisition method.

PBTZ169 and metabolites at highest calibration concentration (2000 ng/mL) did not produce interferences at retention times of ISTD, as shown in Fig 2C. Reciprocally, no relevant interferences emanating from internal standards (at 40 ng/mL) were found at retention time of PBTZ169 and metabolites. MS-based interferences observed for currently known isomeric metabolites (*i*.*e*., presence of multiple chromatographic peaks in the LC-MS/MS trace) were not an issue due to their efficient chromatographic separation (Fig 2A).

As depicted in the Fig 2D, no carryover was observed for the analytes of interest after one blank solvent injection, except for Met oxo. When present, carryover signal for this later metabolite ranged from 20 to 50% of the LLOQ signal with negligible impact on quantitative results.

#### Matrix effect, extraction recovery and process efficiency

Qualitative evaluation of matrix effect. As depicted in S4 Fig, no major *m/z* signal perturbations (*i*.*e*., ion suppression or enhancement) were observed at retention time of the analytes, indicating negligible impact of human plasma components on the ionization process of PBTZ169, currently known active metabolites and ISTD.

Quantitative assessment of matrix effect, extraction recovery and process efficiency. Considering matrix effect, the internal standard–normalized ME (IS-nME) mean values reported in S2 Table for all analytes at 10, 100 and 1000 ng/mL were within ±15%, while precisions (RSD) values were less than 15%. The ISTD selected for PBTZ169 and metabolites were thus found to efficiently control the matrix effect of regular plasma. Yet, oxo and 3-oxo metabolites signals appear to be somewhat affected by lipid components when present at higher concentrations in plasma. Nevertheless the internal standard-normalized extraction recoveries (IS-nER) and process efficiencies (IS-nPE) for all analytes in regular plasma were overall comprised between 80 and 114%, and between 72 and 120%, respectively, with precisions (RSD) lower than 15% at all concentrations. In the limited number of lipemic plasma tested, both extraction recovery and process efficiency were acceptable for PBTZ169 and active metabolites. Data and results for parameters without normalization are also reported in S2 Table.

#### Trueness, precision, accuracy profiles and linearity

Responses functions of PBTZ169 and metabolites were obtained by plotting analyte/internal standard peak area ratios over analyte concentrations in plasma from 0.1 to 2000 ng/mL. Three regression models, *i*.*e*., quadratic weighted 1/x, linear logarithmic and quadratic logarithmic, were evaluated and found to allow a reliable description of response-concentration relationship for the analytes of interest in terms of trueness and precision for back-calculated calibration standard data. For each analyte, the best calibration model was finally selected according to (i) best estimations of trueness and precisions determined by back-calculation of validation standards with daily calibration curve, (ii) the most accurate profile, *i*.*e*., the profile giving the narrowest expectation tolerance intervals (ETIs) and (iii) the largest validation domain (lowest LLOQ). Accordingly, quadratic logarithmic regression model was used for PBTZ169, metabolites 3-OH, 3-oxo and oxo, whereas linear logarithmic regression model was applied to metabolites 1-OH and 2-OH. As reported in Table 2, on the validated range including the LLOQ, trueness (91.5–106.4%), repeatability (0.5–17.2%) and intermediate precision (0.5–17.2%) were in accordance with the recommendations of FDA, and therefore appropriate for quantifying PBTZ169 and its active metabolites in human plasma. According to these results, the LLOQ corresponded to the lowest validation sample concentrations (corresponding hence to the lowest calibration level) and were 0.1 ng/mL for PBTZ169, 0.2 ng/mL for Met 3-OH and 3-oxo, and 0.5 ng/mL for Met 1-OH, 2-OH and oxo. The ULOQ corresponded to the highest validation sample concentration evaluated (*i*.*e*. 1500 ng/mL) for all compounds.

**Table 2:** Trueness, repeatability and intermediate precision determined in human plasma over the validated range for PBTZ169 and active metabolites.

Analytes	Validation Sample Level	Trueness	Precision	
		*Recovery*	*Repeatability*	*Intermediate fidelity*
	ng/mL	%	%	%
*PBTZ169*	0.1	105.0	17.2	17.2
	0.2	99.2	6.1	6.1
	0.5	96.9	2.4	2.4
	1	96.7	2.5	3.5
	2	96.2	1.1	2.1
	150	104.6	0.7	1.5
	750	100.0	1.2	1.5
	1500	99.5	0.9	1.8
*Met*	0.5	100.1	3.4	4.2
*1-OH*	1	106.4	3.6	3.6
	2	92.2	5.8	5.8
	150	95.5	1.9	2.2
	750	99.3	1.3	1.3
	1500	99.1	1.7	1.7
*Met*	0.5	96.8	3.6	4.4
*2-OH*	1	94.6	5.0	5.7
	2	91.5	3.6	3.6
	150	97.2	1.8	2.1
	750	99.8	1.3	2.0
	1500	99.4	1.8	1.9
*Met*	0.2	102.4	8.8	8.8
*3-OH*	0.5	93.0	1.7	6.2
	1	93.7	4.4	5.8
	2	93.1	2.6	4.5
	150	101.5	0.6	2.0
	750	99.6	0.5	0.5
	1500	95.2	1.8	2.5
*Met*	0.2	106.1	7.0	8.4
*3-oxo*	0.5	102.7	4.1	4.7
	1	99.2	3.0	5.6
	2	98.0	5.7	5.7
	150	101.8	4.0	4.0
	750	101.1	1.3	2.3
	1500	97.4	2.7	2.7
*Met*	0.5	102.8	4.1	5.4
*oxo*	1	94.2	2.1	3.7
	2	96.6	1.9	4.0
	150	96.4	2.2	2.2
	750	96.9	2.0	2.0
	1500	96.0	1.5	1.5

**Fig 3:**
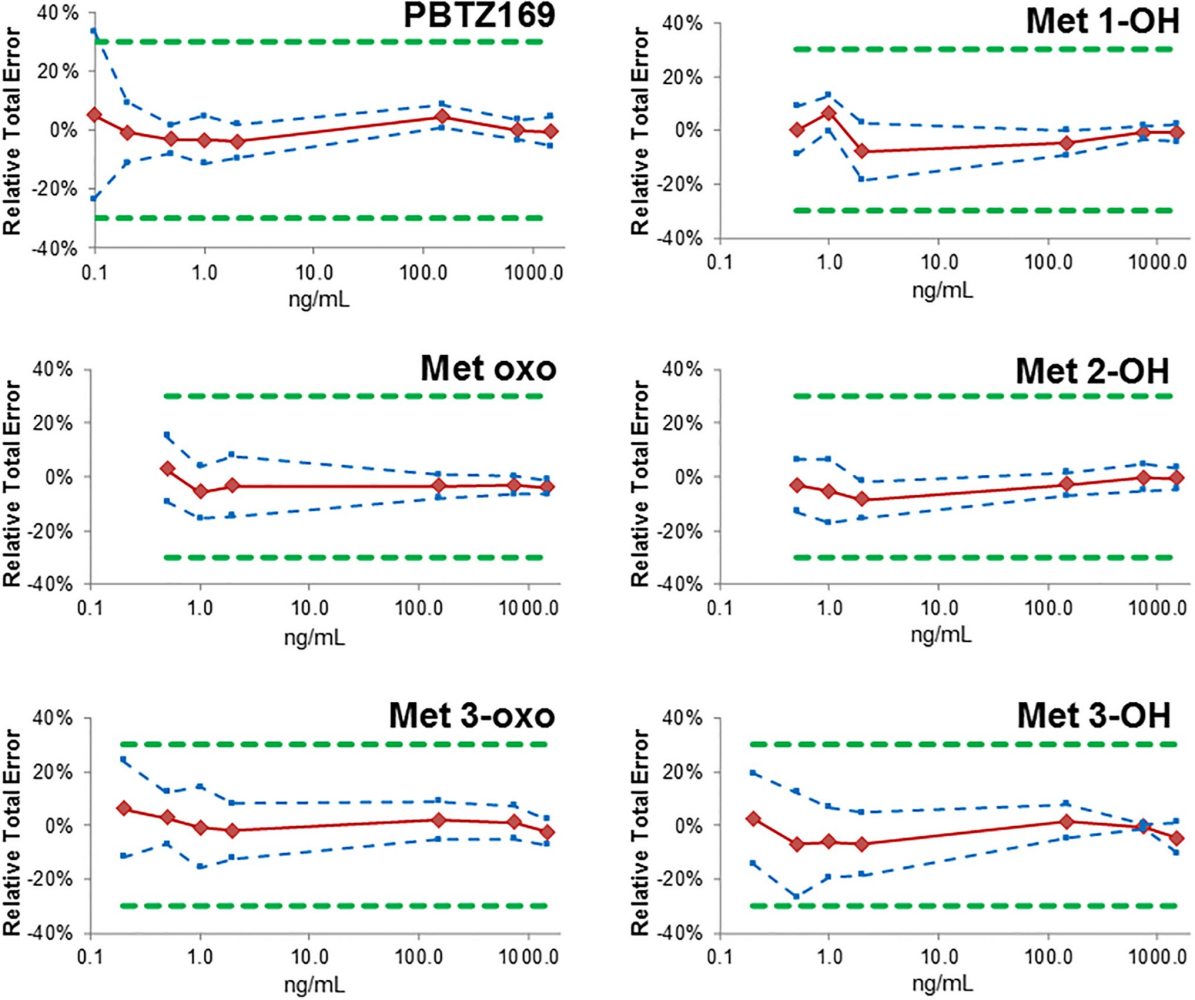
Accuracy profiles over the considered validation domain in plasma of PBTZ169 and its in vitro known active metabolites. Bias (red solid line), upper and lower β-expectation tolerance intervals (β = 90%) (blue dotted lines) and acceptance limits (λ = ± 30%), green dotted lines) are shown.

Accuracy profiles with β-ETIs (Expectation Tolerance Intervals) were determined using a β value of 90%, *i*.*e*., the percentage of future measurements expected to lie within such defined tolerance intervals. As shown in Fig 3, the obtained accuracy profiles were within the acceptance limits of ±30% for biological samples recommended in the recent regulatory guidelines [23, 24, 51], except for PBTZ169 at LLOQ (0.1 ng/ml), which slightly exceeds this allowance with a relative total error of 33.5%.

With this accuracy profiles approach, both LLOQ and ULOQ are graphically determined as the lowest and highest concentration, respectively, across which the β-ETIs span over the acceptance limits expressed in absolute values. This yields in a LLOQ for PBTZ169 of 0.11 ng/mL slightly above (+10%) the LLOQ obtained with classical approach that considers trueness and precision separately. This is however without clinically significant relevance for the intended purpose of pharmacokinetic measurements, and the method has been considered as valid over the entire investigated range (0.1–1500 ng/mL).

To estimate the linearity of trueness, a standard linear regression model was applied to data plots reporting back-calculated validation standard concentrations versus theoretical concentrations. Slopes and intercepts globally ranged from 0.94 to 1.01 and from -3.0 to 6.3, respectively, whereas determination coefficients (R^2^) were all higher than 0.999, indicating satisfactory linearity (S5 Fig).

#### Integrity to dilution and sensitivity

As reported in S3 Table, analyses of plasma samples spiked with PBTZ169 and its five active metabolites above the ULOQ, provide after 10-fold dilution (with blank plasma) concentration values deviating by less than 15% from nominal values with a precision value lower than 4%, demonstrating satisfactory integrity to dilution. Samples containing PBTZ169 and metabolites up to 5000 ng/mL (*i*.*e*. above ULOQ) could be also confidently assayed without prior dilution using the regression models established up to 2000 ng/mL, with less than 15% deviation from nominal values (except for Met 1-OH) and a precision value lower than 3%.

The limits of detection (LOD) for PBTZ169 and five active metabolites in human plasma were ascertained using plasma matrix from three different donors, and correspond to 0.025 ng/mL for PBTZ169, 0.05 ng/mL for metabolites 2-OH, 3-OH and oxo, 0.1 ng/mL for metabolites 1-OH and 3-oxo. To accurately assess the mass spectrometry instrument detection limits (IDL) without the uncertainty due to sample clean-up, analytes were spiked to blank processed plasma sample. IDL values were estimated in three different plasma donors and ranged between 0.9 and 1.3 fg for PBTZ169, 0.5 and 0.6 fg for Met 1-OH, 2-OH and oxo, 0.4 and 0.6 fg for Met 3-OH, and 0.5 and 0.7 fg for Met 3-oxo.

#### Stabilities

Bench-top stability experiments revealed that concentrations of PBTZ169 and active metabolites in processed extracts stored in glass HPLC vials after 24h-storage at +4°C deviated less than ±15% from t_0_ levels, indicating the possibility to re-analyze them within 24h, if necessary (S4 and S5 Tables). After 24h at RT however, both PBTZ169 and its deuterated internal standard were not stable in processed samples, in particular at medium (-30%) and low (-50%) levels. Similarly, all metabolites were highly instable in processed samples when stored at RT.

PBTZ169 was stable in citrated human plasma stored at +4°C as well as at RT for at least 24h. To the contrary, metabolites concentrations in plasma were dramatically reduced after 24h at RT and at +4°C, except Met 1-OH (stable at +4°C). This indicates that collected plasma must be stored at -20°C or preferably at -80°C without delay after blood centrifugation. For both PBTZ169 and metabolites, plasma concentrations after 3 freeze/thaw cycles deviated less than ±15% (±20% for LLOQ concentrations) from concentrations of freshly processed samples, indicating that PBTZ169 and active metabolites were stable in spiked plasma samples after at least three freeze/thaw cycles from -80°C or -20°C to RT.

Stability experiments in citrated whole blood indicate that after withdrawal, blood tubes can be confidently stored temporarily at +4°C or in ice up to 3h prior to centrifugation without noticeable impact on PBTZ169 and metabolites levels. Yet, after 4h at +4°C, Met 3-oxo concentrations deviation exceeded the accepted -15% limit while at RT, both Met 3-oxo and Met 2-OH were found not stable in whole blood.

Long-term stability of PBTZ169 and active metabolites spiked to plasma stored at -20°C and at -80°C was confirmed over 12 months with concentration values globally within ±15% of nominal concentrations (S6 Table).

#### Anticoagulant and serum comparison

The method validation was performed using whole blood and plasma matrices containing citrate as anticoagulant. Yet, calibration established with citrated plasma samples enable to appropriately determine concentrations of PBTZ169 and metabolites in serum and in heparinized or EDTA plasma with overall accuracy and precision within ±15% (±20% at LLOQ) (S7 Table), except for Met 2-OH at low QC level (bias -19%) that possibly reflects analytical inaccuracy for this latter metabolite, rather than anticoagulant impact. Thus, EDTA and heparin are both suitable for blood patient collection and can also be used if necessary for the preparation of calibrators and QC samples.

#### Pharmacologically inactive metabolite amino

The inactive metabolite can be sensitively detected (LLOQ <1 ng/mL) with the developed method and well separated from other metabolites, limiting the risk of analytical interferences (see Analytical method development section).

As shown in Fig 2 and S6 Fig, the method provides a suitable selectivity, a satisfactory control of matrix effects and a negligible carryover also for this inactive Met amino. The internal standard deuterated (-d11) of amino metabolite, available only at the end of the present method validation, was then used throughout all subsequent analyses, also providing robust and accurate quantitative results for Met amino in human plasma by correcting matrix effects, bioanalytical variability and analyte degradation potentially occurring in post-preparative samples (S6 Fig).

#### Identification and stability of the dihydrogenated metabolite (H_2_-PBTZ169) in humans

A recent article from Kloss *et al*. [21] revealed the existence of an additional *in vivo* metabolite formed by the reduction of PBTZ169, leading to the formation of a hydride Meisenheimer complex. When chemically synthetized by the reduction of PBTZ169 with NaBH_4_, a mixture of two position *o*- and *p*- isomers of H_2_-PBTZ169 (S7 Fig) is formed, yet only the *p*-isomer is found *in vivo*.

The main concern about this intriguing Meisenheimer complex of PBTZ169 was its reported poor stability. Kloss *et al*. claimed that H_2_-PBTZ19 was prone to a quick re-oxidization into PBTZ169 when exposed to air, rendering questionable any quantifications of PBTZ169 (and H_2_-PBTZ169) in biological fluids. Kloss *et al*. argued that H_2_-PBTZ19 would likely quickly revert to PBTZ169 during the plasma sample preparation prior to injection into the UPLC column and mass spectrometry analyses. Therefore, we have carried out a comprehensive investigation on the stability of H_2_-PBTZ169 in processed samples stored at 5°C in the autosampler, to ascertain the reliability of concentrations measured with our validated methodology.

The synthetic dihydrogenated compound was used to determine the metabolite-specific MS/MS parameters and for its quantitation, compound 11526102 was used as ISTD. The validated human plasma processing procedure was applied to plasma samples spiked at different concentrations with fresh stock solution of H_2_-PBTZ169. The processed samples were repetitively injected at selected time up to more than 24h after samples preparation and the H_2_-PBTZ169/ISTD peak area ratios were monitored and compared over time (S8 Table). Ratios remained stable over at least 24h, confirming the stability of H_2_-PBTZ169 in processed plasma samples. Moreover in the clinical study, volunteers blood samples were centrifuged within 5–10 min after blood thawing, before plasma collection and immediate storage at -80°C. Thus, with respect to the applied sample collection procedure and the results of our stability study, the measurements of PBTZ169 in clinical plasma samples are unlikely to be flawed to a significant extend by a putative oxidation of H_2_-PBTZ169 metabolite.

#### Pharmacokinetics application

**Fig 4:**
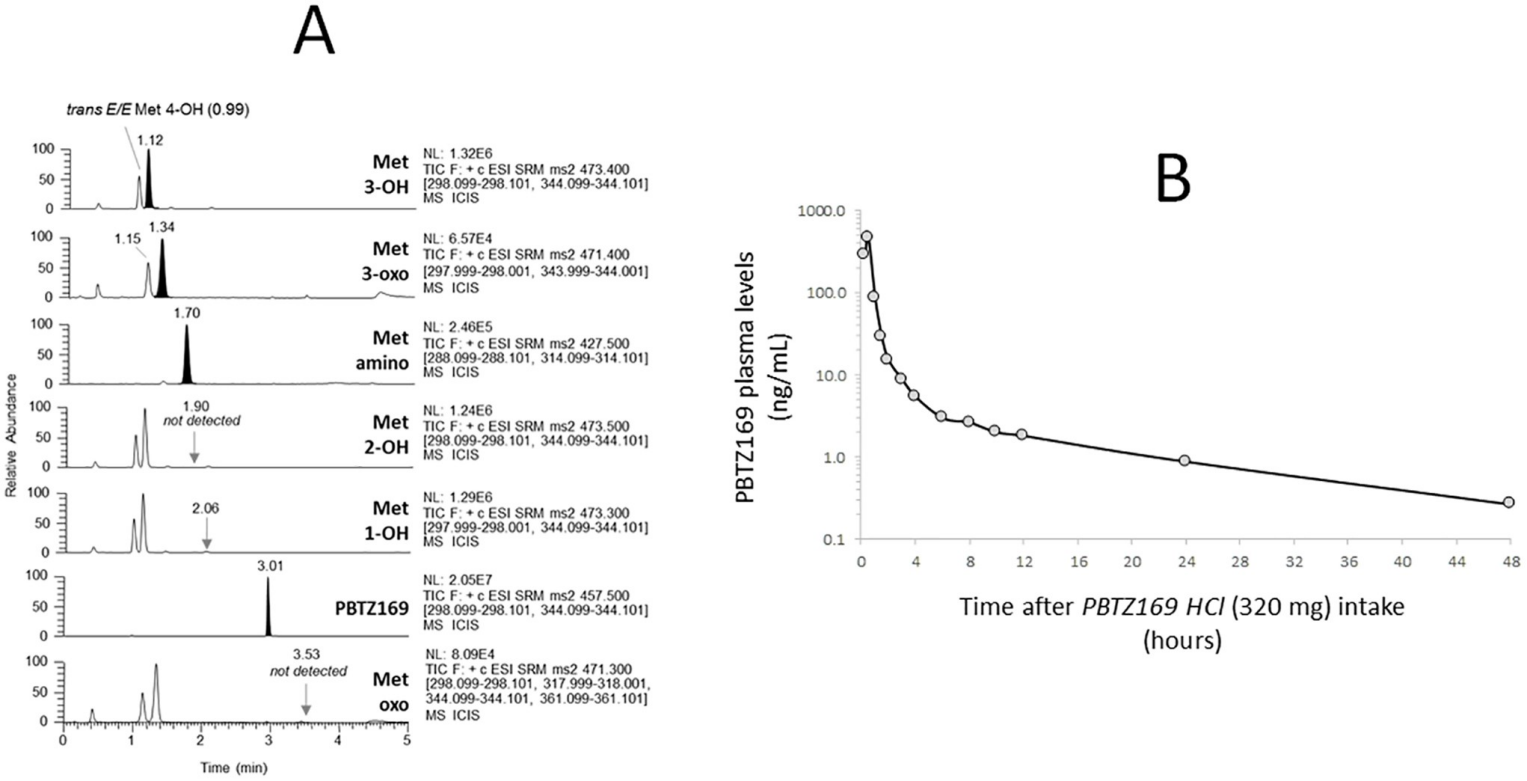
(A) Chromatographic profiles of PBTZ169 and metabolites in plasma collected in an healthy volunteer 30 minutes after the oral intake of 320 mg PBTZ169.HCl. (B) PK profile of PBTZ169 in plasma from the same volunteer, followed for 2 days after the administration of a single dose of 320 mg PBTZ169.HCl.

The present method has been applied for the analyses of plasma from healthy volunteers participating to a phase Ia clinical pharmacokinetic (PK) trial (study name: IM-06-11, *ClinicalTrials*.*gov identifier (NCT number)*: NCT03423030), allowing the multiplex quantification of not only PBTZ169 and its active metabolites levels, but also the inactive metabolite amino, once the labeled ISTD was available. Fig 4A shows an example of multiplex UHPLC-MS/MS profile of a human plasma sample collected 30 min after the intake of a single dose of 320 mg of PBTZ169.HCl.

The concentration measured in this plasma sample is 464 ng/mL, 147 ng/mL, 13 ng/mL, 12 ng/mL and 2 ng/mL for PBTZ169, Met 3-OH, Met 3-oxo, Met amino and Met 1-OH, respectively. The Fig 4B shows the PK profile of PBTZ169 in plasma from the same volunteer followed up to two days post single dose of 320 mg PBTZ169.HCl. Of note, measured plasma levels were multiplied by the factor 1.1 accounting for the blood 10:1 dilution due to citrate solution.

The phase I Met 3-OH was the most abundant *in vivo* active metabolite identified at present, followed by its subsequent oxidative metabolite 3-oxo. Levels of Met 1-OH were globally below 0.5 ng/mL (*i*.*e*. LLOQ value), whereas both metabolites 2-OH and oxo, both identified active *in vitro*, were not detected *in vivo*. Of interest, the plasma concentrations of the metabolite H_2_-PBTZ169 were overall much higher than the parent drug PBTZ169 (S7 Fig) in volunteers, with an estimated peak concentration of 465 ng/mL identified 1 hour after the intake of a single dose of 320 mg of PBTZ169.HCl (same healthy volunteer as Fig 4, for example), with also, parallel final elimination slopes (S7 Fig). PK data used for Fig 4 and S7 Fig are given in S9 Table.

As shown in Fig 4, the targeted MS/MS acquisition reveals the presence *in vivo* of additional peaks that have been detected at the same *m/z* transitions, but distinct retention times, of known metabolites. The PK evolution (estimated using peak areas) of these chemical species after PBTZ169 administration, suggests they are metabolites of PBTZ169 corresponding to hydroxylated (*m/z* 473 Da at 0.99 min) and oxo metabolites (*m/z* 471 Da at 1.15 min). The hydroxylated metabolite eluted at 0.99 min, before the Met 3-OH, was finally identified as the *trans-*4-OH PBTZ169 (*E/E*, both substituents of the cyclohexyl ring in equatorial (*E*) positions) by co-injection with the synthetic diastereoisomeric mixture 65:35 of *trans-E/E-* and *cis*-*E/A-*4OH PBTZ169. Alternately, the *cis-E/A-*4OH PBTZ169 isomer is present at only extremely low level, if not at all, in humans. Such stereoisomeric assignment was possible since the applied chromatographic program allowed to discriminate and separate the *E/E* and *E/A* diastereoisomers for Met 4-OH, while the synthetic metabolites 3-OH and 2-OH appear both as a single peak. Finally, by analogy with Met 4-OH, the oxo metabolite eluted at 1.15 min before Met 3-oxo, is presumably the Met 4-oxo, but this remains to be formally confirmed. The presence of these additional metabolites at significant amounts in clinical samples, but not in calibration samples, underscores the importance of an efficient chromatographic step prior to the mass spectrometry detection. It is also necessary to carefully ascertain the potential matrix effect impact of these metabolites (*i*.*e*. through ion suppression/enhancement) on some ISTDs not co-eluting with their target metabolites, albeit used to quantify them. With our method, peak areas obtained in spiked and clinical samples for ISTD 11526102 and Met amino-d11 perfectly matched, with an overall RSD below 2%, confirming the reliability of the method for measuring not only PBTZ169 but also six known metabolites in real human plasma samples collected from healthy volunteers.

## Conclusion

A multiplex assay including simplified plasma sample preparation together with a rapid, sensitive and robust UHPLC-MS/MS analysis was developed and extensively validated for quantifying the anti-tuberculosis drug candidate PBTZ169 (Macozinone) and its known active metabolites in human plasma. This methodology has been successfully applied for the study of the abundance of PBTZ169 and five active metabolites in samples collected during a safety and pharmacokinetics trial of PBTZ169 in healthy volunteers. Of importance, the Meisenheimer complex H_2_-PBTZ169 was found in our hands more stable than previously reported, and could indeed be satisfactorily quantified in humans, distinctly from PBTZ169. Reciprocally, we have also found that the stated *ex vivo* oxidation of H_2_-PBTZ169 was unlikely to significantly affect the actual PBTZ169 concentrations determined in clinical samples with the proposed assay. Complementary validation procedures are currently performed to integrate into the current multiplex assay the formal quantification of the active metabolite *trans-E/E-*4-OH of PBTZ169, as well the pharmacologically inactive amino metabolite of PBTZ169, the bioreductive product of PBTZ169 that occurs *in vivo*.

## Supporting information

S1 Table Physico-chemical properties (pKa, logP and logD) of PBTZ169 and known metabolites. (DOCX)

S2 Table Matrix effect, extraction recovery and process efficiency assessment. (DOCX)

S3 Table Integrity to dilution. (DOCX)

S4 Table Stability for thaw/freeze cycles. (DOCX)

S5 Table Bench-top stability of PBTZ169 and active metabolites under different storage conditions. (DOCX)

S6 Table Medium-Long term stability in spiked plasma. (DOCX)

S7 Table Anticoagulants and serum comparison. (DOCX)

S8 Table Stability of H2-PBTZ169 metabolite in processed samples. (DOCX)

S9 Table PK concentrations of PBT169 and H2-PBTZ169 metabolite in human plasma. (DOCX)

S1 Fig Qualitative monitoring of representative endogenous phospholipids in human plasma. (DOCX)

S2 Fig Product ion spectra of PBTZ169 and known metabolites. (DOCX)

S3 Fig Optimization of ESI source parameters for PBTZ169 and known metabolites. (DOCX)

S4 Fig Qualitative evaluation of matrix effect for PBTZ169, active metabolites and internal standards. (DOCX)

S5 Fig Linearity of trueness obtained for PBTZ169 and active metabolites in human plasma. (DOCX)

S6 Fig Investigations for inactive amino metabolite. (DOCX)

S7 Fig Structure and pharmacokinetics of metabolite H_2_-PBTZ169. (DOCX)

## Abbreviations

ACN: acetonitrile
DMSO: dimethyl sulfoxide
DT: dwell time
EMEA: European Medicines Agency
ER: extraction recovery
ESI: electrospray ionization
ETI: expectation tolerance interval
FA: formic acid
FDA: Food and Drug Administration
FP6: Sixth Framework Programme
FP7: Seventh Framework Programme
FWHM: full width at half maximum
IDL: instrument detection limit
IS-nER: internal standard normalized extraction recovery
IS-nME: internal standard normalized matrix effect
IS-nPE: internal standard normalized process efficiency
ISTD: internal standard
LLOQ: lower limit of quantification
LOD: limit of detection
MDR-TB: multi-drug resistant tuberculosis
ME: matrix effect
MeOH: methanol
Met: metabolite
MIC: minimum inhibitory concentration
MM4TB: More Medicines for Tuberculosis
NM4TB: New Medicines for Tuberculosis
PE: process efficiency
PP: polypropylene
Q: quadrupole/MS
QC: quality control
QqQ/MS: triple-quadrupole mass spectrometer
RPM: revolutions per minute
RSD: relative standard deviation
RT: room temperature
S/N: signal-to-noise ratio
SFSTP: Société Française des Sciences et Techniques Pharmaceutiques
SRM: selected reaction monitoring
TB: Tuberculosis
TIC: total ion current
t_R_: retention time
UHPLC-MS/MS: ultra-high-pressure liquid chromatography-tandem mass spectrometry
ULOQ: upper limit of quantification
WHO: World Health Organization
WS: working solution
