# Supplementary material for: Development and validation of a multiplex UHPLC-MS/MS method for the determination of the investigational antibiotic against multi-resistant tuberculosis macozinone (PBTZ169) and five active metabolites in human plasma
Source: PLoS One. 2019 May 31;14(5):e0217139. doi: 10.1371/journal.pone.0217139 (PMC6544242; doi:10.1371/journal.pone.0217139)

S1 Table

Physico-chemical properties (pKa, logP and logD) of PBTZ169 and known metabolites (ChemAxon predicted values, <https://chemicalize.com>, accessed 2017/11).


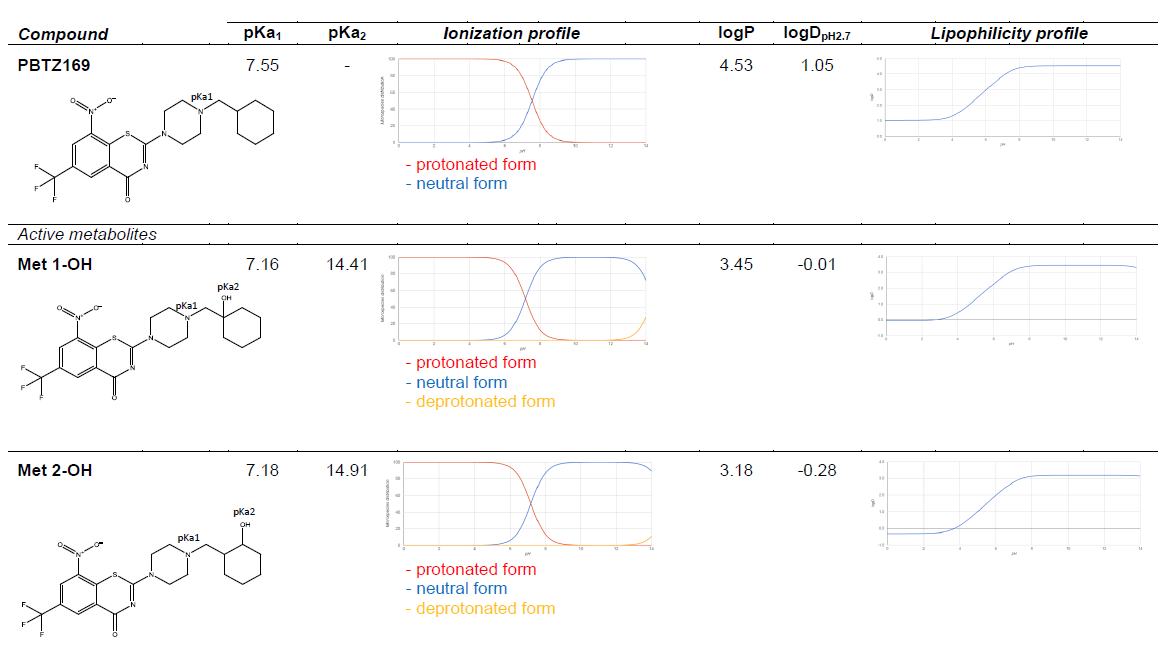

Supplement: S1 Table — (DOCX) [file pone.0217139.s001.docx]
