# Supplementary material for: Development and validation of a multiplex UHPLC-MS/MS method for the determination of the investigational antibiotic against multi-resistant tuberculosis macozinone (PBTZ169) and five active metabolites in human plasma
Source: PLoS One. 2019 May 31;14(5):e0217139. doi: 10.1371/journal.pone.0217139 (PMC6544242; doi:10.1371/journal.pone.0217139)
Supplement: S2 Table — (DOCX) [file pone.0217139.s002.docx]

S2 Table

**Internal Standard normalized Matrix Effect (IS-nME), extraction recovery (IS-nER) and process efficiency (IS-nPE) in regular and lipemic human for PBTZ169 and active metabolites.**

| **Analytes** | **QC Level** | **Matrix Effect**  **(IS-nME)** | | | **Extraction Recovery**  **(IS-nRE)** | | | **Process Efficiency**  **(IS-nPE)** | | |
| --- | --- | --- | --- | --- | --- | --- | --- | --- | --- | --- |
|  |  | Regular  *n*=10  Bias % (RSD) | | Lipemic  *n*=4  Bias % (RSD) | Regular  *n*=10  Recovery % (RSD) | | Lipemic  *n*=4  Recovery %  (RSD) | Regular  *n*=10  Recovery % (RSD) | | Lipemic  *n*=4  Recovery % (RSD) |
|  |  |  | |  |  |  |  |  |  |  |
| PBTZ169 | L | 1 (5) | | 6 (2) | 93 (7) | | 95 (5) | 94 (7) | | 101 (5) |
|  | M | 1 (10) | | 8 (6) | 92 (6) | | 90 (6) | 93 (6) | | 98 (6) |
|  | H | -3 (7) | | 1 (4) | 89 (5) | | 94 (3) | 87 (5) | | 95 (3) |
|  |  |  |  |  |  |  |  |  |  |  |
| Met | L | -6 (8) | | 0 (8) | 92 (10) | | 99 (4) | 87 (10) | | 98 (4) |
| 1-OH | M | -3 (3) | | 1 (6) | 95 (5) | | 95 (1) | 92 (5) | | 96 (1) |
|  | H | 3 (4) | | 5 (2) | 95 (4) | | 95 (3) | 97 (4) | | 99 (3) |
|  |  |  | |  |  | |  |  | |  |
| Met | L | -6 (10) | | -2 (9) | 86 (12) | | 96 (5) | 81 (12) | | 94 (5) |
| 2-OH | M | -4 (3) | | -1 (7) | 91 (5) | | 93 (1) | 87 (5) | | 92 (1) |
|  | H | 2 (4) | | 2 (2) | 93 (4) | | 94 (3) | 95 (4) | | 95 (3) |
|  |  |  | |  |  | |  |  | |  |
| Met | L | -15 (4) | | -4 (7) | 85 (11) | | 96 (3) | 72 (11) | | 92 (3) |
| 3-OH | M | -8 (7) | | 1 (5) | 92 (5) | | 95 (1) | 85 (5) | | 96 (1) |
|  | H | -2 (3) | | 2 (1) | 96 (5) | | 94 (4) | 94 (5) | | 96 (4) |
|  |  |  | |  |  | |  |  | |  |
| Met | L | -6 (11) | | 0 (8) | 82 (12) | | 95 (4) | 78 (12) | | 95 (4) |
| 3-oxo | M | -4 (5) | | 4 (6) | 90 (7) | | 92 (1) | 86 (7) | | 96 (1) |
|  | H | 15 (5) | | 23 (2) | 88 (8) | | 93 (4) | 101 (8) | | 114 (4) |
|  |  |  |  |  |  |  |  |  |  |  |
| Met | L | 7 (8) | | 15 (5) | 80 (7) | | 91 (7) | 86 (7) | | 105 (7) |
| oxo | M | 13 (5) | | 18 (2) | 81 (6) | | 89 (5) | 91 (6) | | 105 (5) |
|  | H | 13 (6) | | 16 (2) | 87 (5) | | 92 (3) | 98 (5) | | 107 (3) |
|  |  |  |  |  |  |  |  |  |  |  |

**Data for Matrix Effect (ME), Extraction Recovery (ER) and Process Efficiency (PE) assessment**

(A) Free matrix sample, (B) Post-extraction spiked plasma, (C) Pre-extraction spiked plasma

**PBTZ169**

*Regular plasma*

*Lipemic plasma*

**Met oxo**

*Regular plasma*

*Lipemic plasma*

**Met 1-OH**

*Regular plasma*

*Lipemic plasma*

**Met 2-OH**

*Regular plasma*

*Lipemic plasma*

**Met 3-OH**

*Regular plasma*

*Lipemic plasma*

**Met 3-oxo**

*Regular plasma*

*Lipemic plasma*
