# Supplementary material for: Development and validation of a multiplex UHPLC-MS/MS method for the determination of the investigational antibiotic against multi-resistant tuberculosis macozinone (PBTZ169) and five active metabolites in human plasma
Source: PLoS One. 2019 May 31;14(5):e0217139. doi: 10.1371/journal.pone.0217139 (PMC6544242; doi:10.1371/journal.pone.0217139)
Supplement: S3 Table — (DOCX) [file pone.0217139.s003.docx]

S3 Table

**Integrity to dilution**

Results obtained for 10-fold diluted plasma samples (5000 ng/mL) and for extrapolation of concentrations using the validated regression models.

| **Analytes** | **Excessive**  **nominal concentration** | **Back- calculated concentration**  **diluted sample^a^** | | **Deviation from nominal value** | **Extrapolated concentration** | | **Deviation from nominal value** |
| --- | --- | --- | --- | --- | --- | --- | --- |
|  |  | Mean^b^ | RSD^b^ |  | Mean^c^ | RSD^c^ |  |
|  | *ng/mL* | *ng/mL* | % | % | *ng/mL* | % | % |
| *PBTZ169* | 5000 | 506.1 | 4 | 1 | 4653.2 | 1 | -7 |
| *Met 1-OH* | 5000 | 486.6 | 1 | -3 | 4140.0 | 2 | -17 |
| *Met 2-OH* | 5000 | 491.1 | 1 | -2 | 5069.4 | 3 | 1 |
| *Met 3-OH* | 5000 | 501.0 | 1 | 0 | 4361.4 | 2 | -13 |
| *Met 3-oxo* | 5000 | 524.1 | 1 | 5 | 4472.4 | 2 | -11 |
| *Met oxo* | 5000 | 490.5 | 2 | -2 | 4614.9 | 2 | -8 |
| ^a^ 10-folds diluted sample  ^b^ *n*=6  ^c^ *n*=4 | | | | | | | |
