# Supplementary material for: Development and validation of a multiplex UHPLC-MS/MS method for the determination of the investigational antibiotic against multi-resistant tuberculosis macozinone (PBTZ169) and five active metabolites in human plasma
Source: PLoS One. 2019 May 31;14(5):e0217139. doi: 10.1371/journal.pone.0217139 (PMC6544242; doi:10.1371/journal.pone.0217139)
Supplement: S4 Table — (DOCX) [file pone.0217139.s004.docx]

S4 Table

**Stability for thaw/freeze cycles**

Stability of a set of calibrators after 3 thaw/freeze cycles (storage at -20°C).

|  | Analyte | | | | | |
| --- | --- | --- | --- | --- | --- | --- |
| Concentration | *PBTZ169* | *Met oxo* | *Met 1-OH* | *Met 2-OH* | *Met 3-OH* | *Met 3-oxo* |
| *ng/mL* | % from freshly processed samples  (n=2) | | | | | |
|  |  |  |  |  |  |  |
| 0.1 | 19 |  |  |  |  |  |
| 0.2 | 11 |  |  |  | 3 | 6 |
| 0.5 | -12 | -11 | -15 | -16 | -14 | -14 |
| 1 | -4 | -12 | -9 | -6 | -13 | -2 |
| 2 | -3 | -6 | -4 | -6 | -10 | -10 |
| 50 | 7 | -10 | -1 | -4 | -8 | -6 |
| 500 | -4 | -8 | -1 | -3 | -6 | -4 |
| 1000 | -10 | -3 | 0 | 0 | -1 | 0 |
| 2000 | -1 | -3 | -1 | -2 | -2 | -1 |
|  |  |  |  |  |  |  |
