# Supplementary material for: Development and validation of a multiplex UHPLC-MS/MS method for the determination of the investigational antibiotic against multi-resistant tuberculosis macozinone (PBTZ169) and five active metabolites in human plasma
Source: PLoS One. 2019 May 31;14(5):e0217139. doi: 10.1371/journal.pone.0217139 (PMC6544242; doi:10.1371/journal.pone.0217139)
Supplement: S5 Table — (DOCX) [file pone.0217139.s005.docx]

S5 Table

**Bench-top stability of PBTZ169 and active metabolites under different storage conditions**

|  |  | Processed  extract sample | | | |  |  | Plasma | |  |  | Whole blood | | | | | | | | |  |  | Plasma  Freeze/thaw | |
| --- | --- | --- | --- | --- | --- | --- | --- | --- | --- | --- | --- | --- | --- | --- | --- | --- | --- | --- | --- | --- | --- | --- | --- | --- |
|  |  | 4°C | | | RT |  |  | 4°C | RT |  |  | ice | | | 4°C | | | RT | | |  |  | -20°C ^a^ | -80°C |
|  |  | 24h | | | |  |  | 24h | |  |  | 1h | 2h | 4h | 1h | 2h | 4h | 1h | 2h | 4h |  |  | 3 cycles | |
| Compound | Level |  |  | % from t_0_ | | | | | | | | | | | | | | | | |  |  | % from freshly  prepared samples | |
|  |  |  | | |  |  |  |  |  |  |  |  |  |  |  |  |  |  |  |  |  |  |  |  |
| PBTZ169 | L | 5 | | | -3 |  |  | 6 | -9 |  |  | 1 | 0 | -1 | 2 | 2 | -1 | -1 | -3 | -7 |  |  | -3 | 5 |
|  | M | -7 | | | 2 |  |  | 1 | -4 |  |  |  |  |  |  |  |  |  |  |  |  |  | 7 | -3 |
|  | H | -15 | | | -8 |  |  | -8 | -9 |  |  | 3 | -1 | 0 | 1 | -3 | -2 | 0 | -4 | -3 |  |  | -10 | -7 |
|  |  |  | | |  |  |  |  |  |  |  |  |  |  |  |  |  |  |  |  |  |  |  |  |
|  |  |  | | |  |  |  |  |  |  |  |  |  |  |  |  |  |  |  |  |  |  |  |  |
| Met 1-OH | L | -12 | | | -55 |  |  | -14 | -59 |  |  | 4 | 1 | -1 | 3 | -2 | 0 | -1 | -4 | -9 |  |  | -4 | -4 |
|  | M | -9 | | | -33 |  |  | -9 | -37 |  |  |  |  |  |  |  |  |  |  |  |  |  | -1 | -3 |
|  | H | -2 | | | -12 |  |  | -7 | -12 |  |  | 2 | 0 | -2 | -1 | -3 | -4 | -4 | -6 | -11 |  |  | 0 | -2 |
|  |  |  | | |  |  |  |  |  |  |  |  |  |  |  |  |  |  |  |  |  |  |  |  |
|  |  |  | | |  |  |  |  |  |  |  |  |  |  |  |  |  |  |  |  |  |  |  |  |
| Met 2-OH | L | -16 | | | -61 |  |  | -31 | -82 |  |  | 1 | -1 | -3 | 1 | -1 | -2 | -10 | -17 | -30 |  |  | -6 | -8 |
|  | M | -10 | | | -39 |  |  | -21 | -62 |  |  |  |  |  |  |  |  |  |  |  |  |  | -4 | -4 |
|  | H | -4 | | | -18 |  |  | -11 | -27 |  |  | 2 | -2 | -3 | -2 | -5 | -6 | -9 | -15 | -26 |  |  | 0 | -4 |
|  |  |  | | |  |  |  |  |  |  |  |  |  |  |  |  |  |  |  |  |  |  |  |  |
|  |  |  | | |  |  |  |  |  |  |  |  |  |  |  |  |  |  |  |  |  |  |  |  |
| Met 3-OH | L | -14 | | | -59 |  |  | -31 | -84 |  |  | 6 | 7 | 4 | 8 | 7 | 12 | 12 | 12 | 15 |  |  | -10 | -7 |
|  | M | -9 | | | -36 |  |  | -26 | -66 |  |  |  |  |  |  |  |  |  |  |  |  |  | -8 | -5 |
|  | H | -2 | | | -14 |  |  | -15 | -23 |  |  | 5 | 4 | 4 | 5 | 1 | 6 | 8 | 9 | 1 |  |  | -1 | -4 |
|  |  |  | | |  |  |  |  |  |  |  |  |  |  |  |  |  |  |  |  |  |  |  |  |
|  |  |  | | |  |  |  |  |  |  |  |  |  |  |  |  |  |  |  |  |  |  |  |  |
| Met 3-oxo | L | -14 | | | -58 |  |  | -33 | -85 |  |  | -1 | -5 | -9 | -6 | -9 | -15 | -22 | -32 | -51 |  |  | -10 | -4 |
|  | M | -9 | | | -37 |  |  | -24 | -67 |  |  |  |  |  |  |  |  |  |  |  |  |  | -6 | -4 |
|  | H | -2 | | | -10 |  |  | -11 | -29 |  |  | 0 | -5 | -10 | -9 | -11 | -18 | -22 | -31 | -50 |  |  | 0 | -4 |
|  |  |  | | |  |  |  |  |  |  |  |  |  |  |  |  |  |  |  |  |  |  |  |  |
|  |  |  | | |  |  |  |  |  |  |  |  |  |  |  |  |  |  |  |  |  |  |  |  |
| Met oxo | L | -3 | | | 6 |  |  | -31 | -87 |  |  | 1 | -3 | -1 | 1 | -4 | 0 | -1 | -8 | -6 |  |  | -6 | -6 |
|  | M | 5 | | | 29 |  |  | -25 | -70 |  |  |  |  |  |  |  |  |  |  |  |  |  | -10 | -8 |
|  | H | 4 | | | 25 |  |  | -10 | -26 |  |  | -1 | -3 | -4 | 0 | 1 | 1 | -5 | -5 | -8 |  |  | -3 | -5 |
|  |  |  | | |  |  |  |  |  |  |  |  |  |  |  |  |  |  |  |  |  |  |  |  |
| ^a^ Whole data are reported in supplementary S4 Table. | | | | | | | | | | | | | | | | | | | | | | | | |
