# Supplementary material for: Development and validation of a multiplex UHPLC-MS/MS method for the determination of the investigational antibiotic against multi-resistant tuberculosis macozinone (PBTZ169) and five active metabolites in human plasma
Source: PLoS One. 2019 May 31;14(5):e0217139. doi: 10.1371/journal.pone.0217139 (PMC6544242; doi:10.1371/journal.pone.0217139)
Supplement: S6 Table — (DOCX) [file pone.0217139.s006.docx]

S6 Table

**Medium-Long term stability in spiked plasma**

Stability of PBTZ169 and active metabolites over 12 months in spiked plasma stored at -20°C and -80°C.
