# Supplementary material for: Development and validation of a multiplex UHPLC-MS/MS method for the determination of the investigational antibiotic against multi-resistant tuberculosis macozinone (PBTZ169) and five active metabolites in human plasma
Source: PLoS One. 2019 May 31;14(5):e0217139. doi: 10.1371/journal.pone.0217139 (PMC6544242; doi:10.1371/journal.pone.0217139)
Supplement: S7 Table — (DOCX) [file pone.0217139.s007.docx]

S7 Table

**Anticoagulants and serum comparison**

- Quantification of QCs prepared at 10, 150 and 1500 ng/mL in plasma (with different anticoagulants) and serum by using citrated plasma calibration.

|  | ***QCs*** | *10 ng/mL*  *(n=3)* | | | *150 ng/mL*  *(n=3)* | | | *1500 ng/mL*  *(n=3)* | | |
| --- | --- | --- | --- | --- | --- | --- | --- | --- | --- | --- |
| **PBTZ** | **Anticoagulant** | **Mean** | **RSD** | **Bias** | **Mean** | **RSD** | **Bias** | **Mean** | **RSD** | **Bias** |
|  | citrate | 10.622 | 3% | 6% | 167.696 | 1% | 12% | 1475.161 | 7% | -2% |
|  | heparine | 10.986 | 5% | 10% | 163.583 | 6% | 9% | 1417.843 | 2% | -5% |
|  | serum | 10.879 | 2% | 9% | 158.624 | 5% | 6% | 1377.335 | 3% | -8% |
|  | EDTA | 11.280 | 3% | 13% | 169.425 | 1% | 13% | 1375.875 | 3% | -8% |
| **Met oxo** | citrate | 11.032 | 4% | 10% | 165.393 | 5% | 10% | 1429.366 | 7% | -5% |
|  | heparine | 11.195 | 2% | 12% | 161.842 | 5% | 8% | 1452.857 | 6% | -3% |
|  | serum | 10.630 | 4% | 6% | 161.980 | 4% | 8% | 1432.824 | 5% | -4% |
|  | EDTA | 10.986 | 0% | 10% | 166.959 | 3% | 11% | 1457.627 | 2% | -3% |
| **Met 1-OH** | citrate | 10.046 | 7% | 0% | 152.733 | 7% | 2% | 1506.014 | 7% | 0% |
|  | heparine | 9.551 | 9% | -4% | 145.198 | 11% | -3% | 1519.324 | 10% | 1% |
|  | serum | 9.465 | 3% | -5% | 143.401 | 3% | -4% | 1455.292 | 5% | -3% |
|  | EDTA | 10.175 | 5% | 2% | 155.408 | 4% | 4% | 1532.710 | 3% | 2% |
| **Met 2-OH** | citrate | 8.658 | 7% | -13% | 143.613 | 8% | -4% | 1509.692 | 8% | 1% |
|  | heparine | 8.074 | 11% | -19% | 136.560 | 12% | -9% | 1502.808 | 11% | 0% |
|  | serum | 8.113 | 6% | -19% | 133.556 | 5% | -11% | 1454.094 | 7% | -3% |
|  | EDTA | 8.642 | 5% | -14% | 147.576 | 4% | -2% | 1539.149 | 3% | 3% |
| **Met 3-OH** | citrate | 10.096 | 8% | 1% | 158.742 | 6% | 6% | 1433.239 | 5% | -4% |
|  | heparine | 9.702 | 8% | -3% | 153.634 | 10% | 2% | 1435.939 | 8% | -4% |
|  | serum | 9.644 | 5% | -4% | 151.514 | 4% | 1% | 1394.746 | 3% | -7% |
|  | EDTA | 10.159 | 5% | 2% | 163.841 | 3% | 9% | 1465.997 | 3% | -2% |
| **Met 3-oxo** | citrate | 9.551 | 7% | -4% | 153.533 | 6% | 2% | 1387.558 | 6% | -7% |
|  | heparine | 9.138 | 8% | -9% | 145.418 | 9% | -3% | 1385.258 | 8% | -8% |
|  | serum | 9.217 | 5% | -8% | 147.333 | 4% | -2% | 1356.501 | 5% | -10% |
|  | EDTA | 9.634 | 5% | -4% | 158.913 | 5% | 6% | 1459.292 | 5% | -3% |

- Quantification of plasma calibrators in EDTA calculated with validated citrated plasma calibration

|  | ***CALs*** | *Replicates* | | |  |  |  |
| --- | --- | --- | --- | --- | --- | --- | --- |
| **PBTZ** | **Levels** | **1** | **2** | **3** | **Mean** | **RSD** | **Bias** |
|  | 0.100 | 0.105 | 0.099 | 0.101 | 0.102 | 3% | 2% |
|  | 0.200 | 0.198 | 0.226 | 0.176 | 0.200 | 12% | 0% |
|  | 0.500 | 0.446 | 0.445 | 0.439 | 0.443 | 1% | -11% |
|  | 1.000 | 1.004 | 0.857 | 0.875 | 0.912 | 9% | -9% |
|  | 2.000 | 2.011 | 1.778 | 1.891 | 1.893 | 6% | -5% |
|  | 50.000 | 57.631 | 54.683 | 60.225 | 57.513 | 5% | 15% |
|  | 500.000 | 522.777 | 513.082 | 546.151 | 527.337 | 3% | 5% |
|  | 1000.000 | 983.562 | 996.603 | 982.395 | 987.520 | 1% | -1% |
|  | 2000.000 | 1957.338 | 1826.104 | 1800.626 | 1861.356 | 5% | -7% |
| **Met oxo** | 0.500 | 0.568 | 0.538 | 0.496 | 0.534 | 7% | 7% |
|  | 1.000 | 1.054 | 0.936 | 1.023 | 1.004 | 6% | 0% |
|  | 2.000 | 1.837 | 1.943 | 1.967 | 1.916 | 4% | -4% |
|  | 50.000 | 52.556 | 58.016 | 59.320 | 56.631 | 6% | 13% |
|  | 500.000 | 518.654 | 532.993 | 536.646 | 529.431 | 2% | 6% |
|  | 1000.000 | 1032.978 | 1019.999 | 999.664 | 1017.547 | 2% | 2% |
|  | 2000.000 | 1995.616 | 1914.333 | 1895.063 | 1935.004 | 3% | -3% |
| **Met 1-OH** | 0.500 | 0.583 | 0.620 | 0.650 | 0.618 | 5% | 24% |
|  | 1.000 | 1.077 | 1.156 | 1.216 | 1.150 | 6% | 15% |
|  | 2.000 | 1.899 | 2.098 | 2.100 | 2.032 | 6% | 2% |
|  | 50.000 | 46.427 | 49.545 | 53.224 | 49.732 | 7% | -1% |
|  | 500.000 | 485.413 | 533.811 | 559.919 | 526.381 | 7% | 5% |
|  | 1000.000 | 1009.805 | 1066.653 | 1120.675 | 1065.711 | 5% | 7% |
|  | 2000.000 | 2124.805 | 2068.556 | 2170.022 | 2121.128 | 2% | 6% |
| **Met 2-OH** | 0.500 | 0.415 | 0.475 | 0.507 | 0.466 | 10% | -7% |
|  | 1.000 | 0.775 | 0.947 | 0.906 | 0.876 | 10% | -12% |
|  | 2.000 | 1.500 | 1.788 | 1.714 | 1.667 | 9% | -17% |
|  | 50.000 | 40.177 | 46.026 | 48.813 | 45.005 | 10% | -10% |
|  | 500.000 | 462.299 | 536.020 | 557.532 | 518.617 | 10% | 4% |
|  | 1000.000 | 983.871 | 1087.475 | 1130.637 | 1067.328 | 7% | 7% |
|  | 2000.000 | 2183.594 | 2142.913 | 2247.320 | 2191.276 | 2% | 10% |
| **Met 3-OH** | 0.200 | 0.199 | 0.157 | 0.232 | 0.196 | 19% | -2% |
|  | 0.500 | 0.472 | 0.528 | 0.549 | 0.516 | 8% | 3% |
|  | 1.000 | 0.873 | 0.926 | 0.919 | 0.906 | 3% | -9% |
|  | 2.000 | 1.614 | 2.156 | 1.915 | 1.895 | 14% | -5% |
|  | 50.000 | 50.127 | 54.071 | 57.507 | 53.902 | 7% | 8% |
|  | 500.000 | 502.122 | 533.582 | 558.318 | 531.340 | 5% | 6% |
|  | 1000.000 | 1013.489 | 1051.329 | 1092.618 | 1052.479 | 4% | 5% |
|  | 2000.000 | 2071.138 | 1972.856 | 2058.420 | 2034.138 | 3% | 2% |
| **Met 3-oxo** | 0.200 | 0.201 | 0.239 | 0.267 | 0.236 | 14% | 18% |
|  | 0.500 | 0.489 | 0.579 | 0.531 | 0.533 | 8% | 7% |
|  | 1.000 | 0.827 | 0.968 | 0.951 | 0.916 | 8% | -8% |
|  | 2.000 | 1.640 | 1.927 | 1.906 | 1.824 | 9% | -9% |
|  | 50.000 | 45.693 | 51.445 | 55.452 | 50.863 | 10% | 2% |
|  | 500.000 | 481.031 | 532.399 | 560.798 | 524.743 | 8% | 5% |
|  | 1000.000 | 970.259 | 1073.290 | 1140.336 | 1061.295 | 8% | 6% |
|  | 2000.000 | 2031.899 | 2046.747 | 2164.784 | 2081.143 | 3% | 4% |
