# Supplementary material for: Development and validation of a multiplex UHPLC-MS/MS method for the determination of the investigational antibiotic against multi-resistant tuberculosis macozinone (PBTZ169) and five active metabolites in human plasma
Source: PLoS One. 2019 May 31;14(5):e0217139. doi: 10.1371/journal.pone.0217139 (PMC6544242; doi:10.1371/journal.pone.0217139)
Supplement: S8 Table — (DOCX) [file pone.0217139.s008.docx]

S8 Table

**Stability of H_2_-PBTZ169 metabolite in processed samples**

Variation of H_2_-PBTZ169/internal standard peak area ratios, in function of the time of analysis post-processing of four plasma samples spiked with different H_2_-PBTZ169 concentrations (HPLC vials stored in autosampler at +5°C)

| Time after sample preparation (h) | 3.4 | 6.7 | 10.1 | 13.4 | 16.7 | 20.1 | 23.3 | 26.4 |
| --- | --- | --- | --- | --- | --- | --- | --- | --- |
| Concentration | Deviation from nominal value (%) | | | | | | | |
| 1 ng/mL | -10 | -3 | 2 | -3 | 0 | -4 | -7 | -4 |
| 10 ng/mL | -2 | 0 | 2 | 1 | 1 | 1 | -2 | 1 |
| 50 ng/mL | -2 | 3 | 5 | 3 | 0 | 0 | -3 | -3 |
| 750 ng/mL | 3 | -1 | -2 | -1 | -4 | -5 | -14 | -10 |
