# Supplementary material for: Development and validation of a multiplex UHPLC-MS/MS method for the determination of the investigational antibiotic against multi-resistant tuberculosis macozinone (PBTZ169) and five active metabolites in human plasma
Source: PLoS One. 2019 May 31;14(5):e0217139. doi: 10.1371/journal.pone.0217139 (PMC6544242; doi:10.1371/journal.pone.0217139)
Supplement: S9 Table — (DOCX) [file pone.0217139.s009.docx]

S9 Table

**PK concentrations of PBTZ169 and H2-PBTZ metabolite in human plasma**

Plasma concentrations (ng/mL) of PBTZ169 and its reduced metabolite (H2-PBTZ) at different time points after the intake of a single dose of 320 mg of PBTZ169.HCl in one healthy volunteer.

|  | Plasma concentration  (ng/mL) | |
| --- | --- | --- |
| Time points after PBTZ169 intake | H2-PBTZ | PBTZ169 |
| Pre-dose | 1.0 | 0 |
| 15 min | 53.1 | 284.4 |
| 30 min | 252 | 464 |
| 1 h | 464.5 | 85.1 |
| 1.5 h | 456.1 | 28.3 |
| 2 h | 395.9 | 14.9 |
| 3 h | 299.1 | 8.7 |
| 4 h | 240.4 | 5.3 |
| 6 h | 158.5 | 2.9 |
| 8 h | 144.1 | 2.6 |
| 10 h | 120.3 | 2 |
| 12 h | 107.8 | 1.8 |
| 24 h | 63.8 | 0.9 |
| 48 h | 22.2 | 0.3 |
