# Supplementary material for: Development and validation of a multiplex UHPLC-MS/MS method for the determination of the investigational antibiotic against multi-resistant tuberculosis macozinone (PBTZ169) and five active metabolites in human plasma
Source: PLoS One. 2019 May 31;14(5):e0217139. doi: 10.1371/journal.pone.0217139 (PMC6544242; doi:10.1371/journal.pone.0217139)
Supplement: S1 Fig — (DOCX) [file pone.0217139.s010.docx]

S1 Fig

**Qualitative monitoring of representative endogenous phospholipids in human plasma**

UHPLC-MS/MS analysis of residual phospholipids in human plasma after sample preparation using MeOH by using the developed LC-MS method. Representative phospholipids A, B, C and D were monitored according to reference 42. For all SRM transitions, collision energy was set at 30 eV and RF at 120 V.
