# Supplementary material for: Development and validation of a multiplex UHPLC-MS/MS method for the determination of the investigational antibiotic against multi-resistant tuberculosis macozinone (PBTZ169) and five active metabolites in human plasma
Source: PLoS One. 2019 May 31;14(5):e0217139. doi: 10.1371/journal.pone.0217139 (PMC6544242; doi:10.1371/journal.pone.0217139)

S2 Fig

**Product ion spectra of PBTZ169 and known metabolites**

MS/MS spectra (50 - 500 *m/z*) of PBTZ169 and metabolites were obtained by individual infusion of the analytes at 500 ng/mL in MeOH and by applying energy collision of 30 for PBTZ169 and 25 eV for metabolites. ESI parameters were adapted to low infusion rate (10 µL/min).


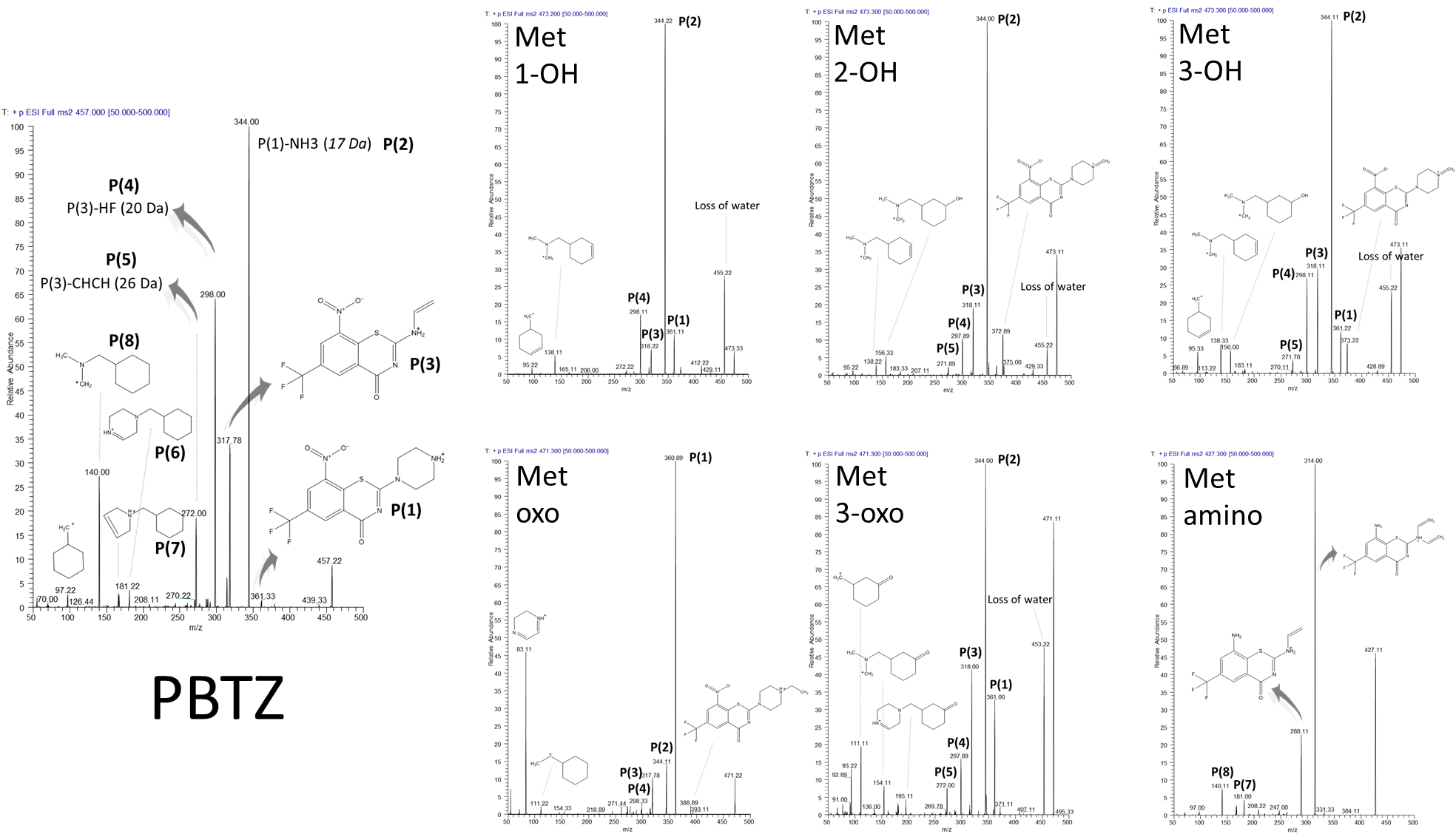

Supplement: S2 Fig — (DOCX) [file pone.0217139.s011.docx]
