# Supplementary material for: Development and validation of a multiplex UHPLC-MS/MS method for the determination of the investigational antibiotic against multi-resistant tuberculosis macozinone (PBTZ169) and five active metabolites in human plasma
Source: PLoS One. 2019 May 31;14(5):e0217139. doi: 10.1371/journal.pone.0217139 (PMC6544242; doi:10.1371/journal.pone.0217139)
Supplement: S3 Fig — (DOCX) [file pone.0217139.s012.docx]

S3 Fig

**Optimization of ESI source parameters for PBTZ169 and known metabolites**

The spray voltage, ion transfer tube and vaporizer gas temperatures as well as sheat, auxillary and sweep ion gas flow rates were optimized by applying the developed LC-MS/MS method and by analysis an extracted (MeOH) plasma sample spiked at 100 ng/mL. The signal (peak area) variation was investigated over the investigated parameters.
