# Supplementary material for: Development and validation of a multiplex UHPLC-MS/MS method for the determination of the investigational antibiotic against multi-resistant tuberculosis macozinone (PBTZ169) and five active metabolites in human plasma
Source: PLoS One. 2019 May 31;14(5):e0217139. doi: 10.1371/journal.pone.0217139 (PMC6544242; doi:10.1371/journal.pone.0217139)

S4 Fig

**Qualitative evaluation of matrix effect for PBTZ169, active metabolites and internal standards**

Individual post-column infusion (10 µL/min) of the analytes (at 500 ng/mL in methanol) directly into MS/MS detector during the chromatographic analysis of blank human plasma from 7 different donors (including two lipemic plasma) and 3 pooled plasma, all processed with pure methanol. Retention times and individual chromatographic LC-MS/MS profiles of PBTZ169, active metabolites and ISTD obtained with the developed method were overlaid for interpretation.


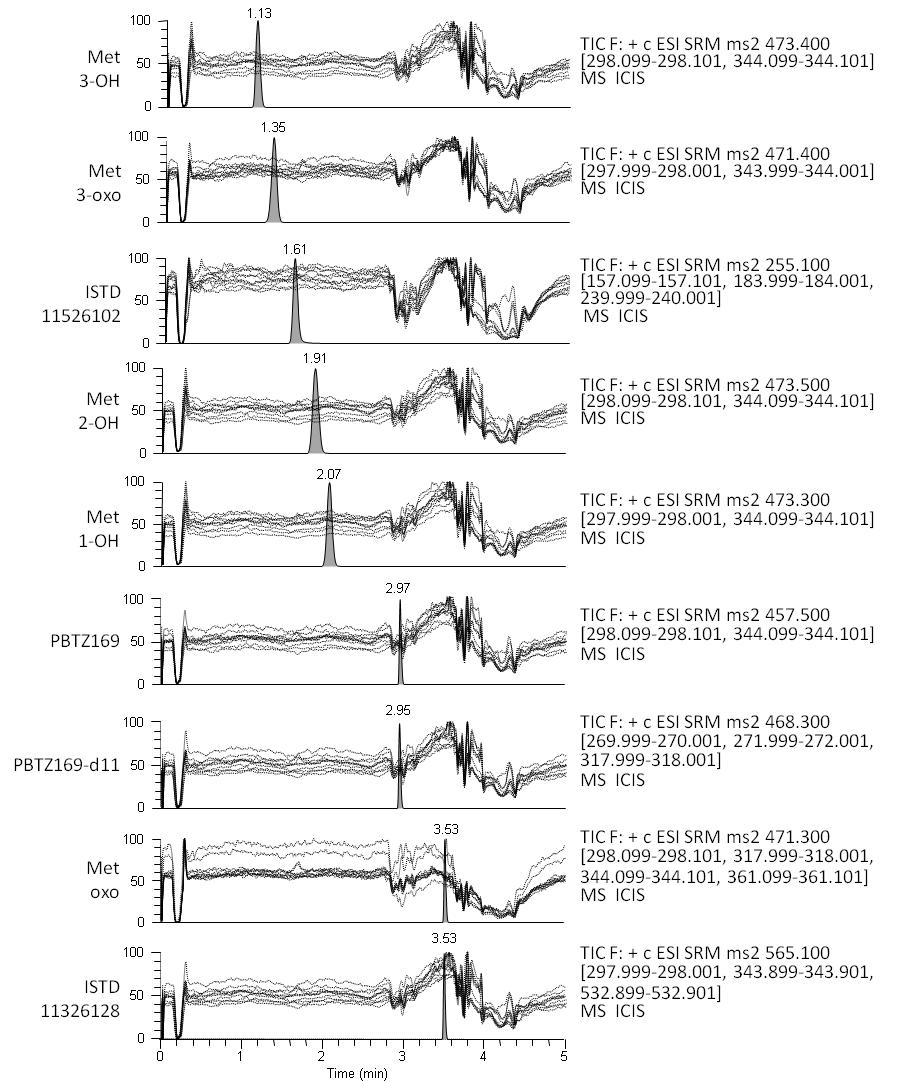

Supplement: S4 Fig — (DOCX) [file pone.0217139.s013.docx]
