# Supplementary material for: Development and validation of a multiplex UHPLC-MS/MS method for the determination of the investigational antibiotic against multi-resistant tuberculosis macozinone (PBTZ169) and five active metabolites in human plasma
Source: PLoS One. 2019 May 31;14(5):e0217139. doi: 10.1371/journal.pone.0217139 (PMC6544242; doi:10.1371/journal.pone.0217139)
Supplement: S5 Fig — (DOCX) [file pone.0217139.s014.docx]

S5 Fig

**Linearity of trueness obtained for PBTZ169 and active metabolites in human plasma**

Linearity obtained reporting back-calculated results obtained during the 3-days validation over nominal concentrations.
