# Supplementary material for: Development and validation of a multiplex UHPLC-MS/MS method for the determination of the investigational antibiotic against multi-resistant tuberculosis macozinone (PBTZ169) and five active metabolites in human plasma
Source: PLoS One. 2019 May 31;14(5):e0217139. doi: 10.1371/journal.pone.0217139 (PMC6544242; doi:10.1371/journal.pone.0217139)
Supplement: S6 Fig — (DOCX) [file pone.0217139.s015.docx]

S6 Fig

**Investigations for inactive amino metabolite**

- Qualitative evaluation of matrix effects for inactive metabolite amino (first chromatogram);
- Chromatographic monitoring of the chemical degradation of this metabolite in DMSO during long-term storage at -20°C with multiple thaw/freeze cycles (from first to fifth chromatogram);
- LC-MS/MS trace of major product of chemical degradation of metabolite amino (sixth chromatogram) and its putative molecular structures (right side); (peak fronting for amino metabolite which evolved during storage to a minor isobaric peak at 1.5 min,)
- LC-MS/MS trace of stable isotopically labeled metabolite amino (-d11) (last chromatogram).

Because of the bioreduction of the nitro group of PBTZ169, the amino metabolite is devoid of anti-TB activity, and its distinct physico-chemical characteristics makes it vulnerable to chemical oxidations in the presence of light. Stock solution of the amino metabolite in DMSO was found to degrade after 6 months of storage at -20°C along with a few freeze/thaw cycles. Visually, the amino metabolite solution turns from colorless to light yellow while chromatographic analysis indicates that in the oxidizing DMSO medium, the amino metabolite (*m/z* 427.5) is progressively converted into a major degradation product eluted at 1.5 min characterized by a precursor ion at *m/z* 443 (see supplementary S6 Figure, that includes the proposed structure of two putative isobaric compounds at *m/z* 443 both characterized by an extended electronic delocalization after amino metabolite oxidation in DMSO). Single-use aliquots of amino metabolite stock solution in DMSO or in ethyl acetate are therefore required to minimize its degradation induced by freeze/thaw cycles.

The medium-long term stability experiments available at present shows that working solutions of pure Met amino in DMSO:MeOH (1:1 *v/v*) are stable over 3 months at -20°C, whereas spiked plasma samples stability has been verified for 1 month at -20°C and -80°C.
