# Supplementary material for: Development and validation of a multiplex UHPLC-MS/MS method for the determination of the investigational antibiotic against multi-resistant tuberculosis macozinone (PBTZ169) and five active metabolites in human plasma
Source: PLoS One. 2019 May 31;14(5):e0217139. doi: 10.1371/journal.pone.0217139 (PMC6544242; doi:10.1371/journal.pone.0217139)

S7 Fig

**Structure and pharmacokinetics of metabolite H_2_-PBTZ169**

(A) Structure of the two ortho- and para- isomers of metabolite H2-PBTZ169. Only the para-isomer is found *in vivo*.

(B) Overlaid pharmacokinetics plots of PBTZ169 and H_2_-PBTZ169 in plasma from the same volunteer, followed for 2 days after the administration of a single dose of 320 mg PBTZ169.HCl.


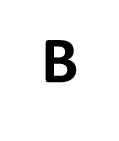

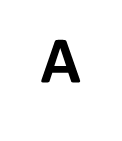

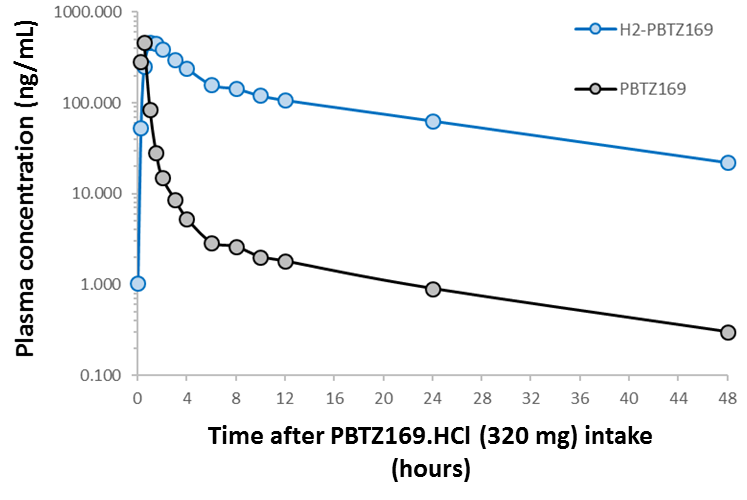

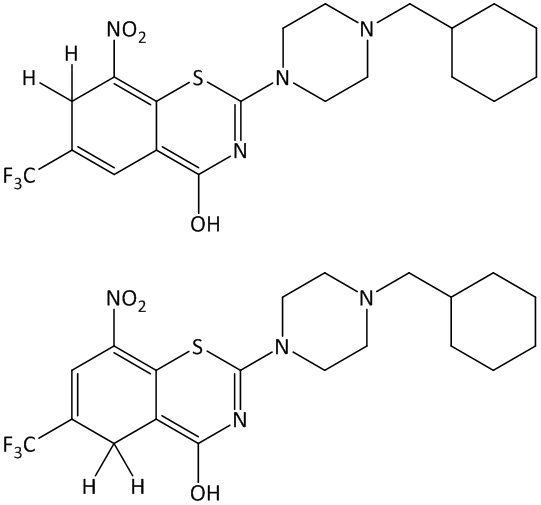

Supplement: S7 Fig — (DOCX) [file pone.0217139.s016.docx]
